# Gene expression profiles and signaling mechanisms in α_2B_-adrenoceptor-evoked proliferation of vascular smooth muscle cells

**DOI:** 10.1186/s12918-017-0439-8

**Published:** 2017-06-28

**Authors:** Anna Huhtinen, Vesa Hongisto, Asta Laiho, Eliisa Löyttyniemi, Dirk Pijnenburg, Mika Scheinin

**Affiliations:** 1 Department of Pharmacology, Drug Development and Therapeutics, Institute of Biomedicine, University of Turku, Kiinamyllynkatu 10, FI-20520 Turku, Finland; 2 Toxicology Division, Misvik Biology Oy, Turku, Finland; 3 Turku Centre for Biotechnology, University of Turku and Åbo Akademi University, Turku, Finland; 4 Department of Biostatistics, Department of Clinical Medicine, University of Turku, Turku, Finland; 5 PamGene International BV, Wolvenhoek 10, 5211HH s’Hertogenbosch, The Netherlands; 6 Unit of Clinical Pharmacology, Turku University Hospital, Turku, Finland

**Keywords:** Alpha2-adrenoceptor, A7r5 cell line, Vascular smooth muscle cell, DNA microarray, Kinase inhibitor, Kinase activity profiling, Functional analysis, Pathway analysis

## Abstract

**Background:**

α_2_-adrenoceptors are important regulators of vascular tone and blood pressure. Regulation of cell proliferation is a less well investigated consequence of α_2_-adrenoceptor activation. We have previously shown that α_2B_-adrenoceptor activation stimulates proliferation of vascular smooth muscle cells (VSMCs). This may be important for blood vessel development and plasticity and for the pathology and therapeutics of cardiovascular disorders. The underlying cellular mechanisms have remained mostly unknown. This study explored pathways of regulation of gene expression and intracellular signaling related to α_2B_-adrenoceptor-evoked VSMC proliferation.

**Results:**

The cellular mechanisms and signaling pathways of α_2B_-adrenoceptor-evoked proliferation of VSMCs are complex and include redundancy. Functional enrichment analysis and pathway analysis identified differentially expressed genes associated with α_2B_-adrenoceptor-regulated VSMC proliferation. They included the upregulated genes Egr1, F3, Ptgs2 and Serpine1 and the downregulated genes Cx3cl1, Cav1, Rhoa, Nppb and Prrx1. The most highly upregulated gene, Lypd8, represents a novel finding in the VSMC context. Inhibitor library screening and kinase activity profiling were applied to identify kinases in the involved signaling pathways. Putative upstream kinases identified by two different screens included PKC, Raf-1, Src, the MAP kinases p38 and JNK and the receptor tyrosine kinases EGFR and HGF/HGFR. As a novel finding, the Src family kinase Lyn was also identified as a putative upstream kinase.

**Conclusions:**

α_2B_-adrenoceptors may mediate their pro-proliferative effects in VSMCs by promoting the activity of bFGF and PDGF and the growth factor receptors EGFR, HGFR and VEGFR-1/2. The Src family kinase Lyn was also identified as a putative upstream kinase. Lyn is known to be expressed in VSMCs and has been identified as an important regulator of GPCR trafficking and GPCR effects on cell proliferation. Identified Ser/Thr kinases included several PKC isoforms and the β-adrenoceptor kinases 1 and 2. Cross-talk between the signaling mechanisms involved in α_2B_-adrenoceptor-evoked VSMC proliferation thus appears to involve PKC activation, subsequent changes in gene expression, transactivation of EGFR, and modulation of kinase activities and growth factor-mediated signaling. While many of the identified individual signals were relatively small in terms of effect size, many of them were validated by combining pathway analysis and our integrated screening approach.

**Electronic supplementary material:**

The online version of this article (doi:10.1186/s12918-017-0439-8) contains supplementary material, which is available to authorized users.

## Background

The α_2_-adrenoceptors, a subclass of the family of G-protein coupled receptors (GPCRs), are targets for cardiovascular drug development because they mediate important actions of noradrenaline and adrenaline in the regulation of vascular tone and blood pressure. The vascular effects of α_2_-adrenoceptor agonists are complex, as they reflect the results of the interplay between centrally mediated sympatholytic effects and pre- and postsynaptic α_2_-adrenoceptor activation in peripheral tissues [1]. The roles of the different α_2_-adrenoceptor subtypes in short-term cardiovascular regulation are relatively well known [2–6]. Indeed, central α_2A_-adrenoceptors are currently employed as targets of clonidine-like antihypertensive drugs [7], but vascular α_2_-adrenoceptors have so far not been exploited as cardiovascular drug targets.

Regulation of cell proliferation is a less well investigated consequence of α_2_-adrenoceptor activation. It has been reported to enhance the proliferation of many different breast cancer cell lines [8–11], primary rat proximal tubule cells [12], opossum kidney cells [13] and Chinese hamster lung fibroblasts [14]. Also inhibition of cholangiocarcinoma and pheochromocytoma cell proliferation by α_2_-adrenoceptor agonists has been reported [15, 16]. In many of these studies, the investigated cells expressed all three α_2_-adrenoceptor subtypes, making it impossible to specify the subtype(s) involved. Some of the studies indicated that enhanced proliferation was caused by activation of α_2A_-adrenoceptors [14] or α_2B_-adrenoceptors [11, 12], whereas inhibition of proliferation was ascribed to α_2C_-adrenoceptors [16]. However, little is known about the effects of α_2_-adrenoceptor activation on the proliferation of vascular smooth muscle cells (VSMCs). Our own previous results indicated that activation of α_2B_-adrenoceptors would have prominent stimulatory effects on the proliferation of cultured A7r5 rat VSMCs [17].

VSMC proliferation and differentiation are essential physiological processes in vascular development and plasticity. Phenotypic switching from differentiated to proliferative VSMCs includes reduced expression of contractile proteins, and increased expression of inflammatory cytokines, proteases and extracellular matrix proteins, and is involved in the development of many major cardiovascular diseases, such as atherosclerosis and hypertension. Increased VSMC proliferation also contributes to restenosis after coronary bypass or angioplasty, limiting the long-term success of these clinical interventions. Improved understanding of the mechanisms regulating VSMC proliferation may therefore guide the development of new therapies [18–20]. In healthy vessels, VSMCs have very low rates of proliferation, but cell proliferation is stimulated by injury or insults to the vessel wall. Pathological loss of quiescence is triggered by release of mitogens from platelets and VSMCs. They activate signaling pathways that stimulate expression of cell-cycle genes. Inactivation of signals that normally repress VSMC proliferation is also required [19, 21, 22].

We have previously demonstrated that activation of α_2B_-adrenoceptors with the selective agonist dexmedetomidine potently increases the proliferation of cultured A7r5 cells, a commonly employed model of VSMCs [17]. However, the underlying cellular mechanisms and signal transduction pathways have remained unknown. We now employed three different screening assays to investigate changes in gene expression, signaling pathways and kinase activation profiles related to α_2B_-adrenoceptor-evoked VSMC proliferation. Another purpose of this study was to evaluate the suitability of DNA microarrays, kinase/phosphatase inhibitor library screening and kinase activity profiling assays for the investigation of these mechanisms.

## Methods

### Materials

A7r5 cells were obtained from the American Type Culture Collection (ATCC; Manassas, VA, USA). Fetal bovine serum was from PAA Laboratories GmbH (Pasching, Austria), trypsin-Versene® solution was from Lonza (Basel, Switzerland). M-PER Mammalian Extraction Buffer, Pierce™ BCA protein assay kit and Halt™ phosphatase and protease inhibitors were from Thermo Fisher Scientific (Waltham, MA, USA). Dulbecco’s modified Eagle’s medium (DMEM), Geneticin (G418 disulphate salt solution), trypsin, EDTA and DMSO were from Sigma Aldrich (St. Louis, MO, USA). Dexmedetomidine was a kind gift from Orion Pharma (Turku, Finland). Oligonucleotide primers were from Oligomer (Helsinki, Finland). Other chemicals and reagents were obtained from commercial suppliers.

### Cell culture

A7r5-α_2B_ cells transfected to stably express the human α_2B_-adrenoceptor were cultured and maintained as described previously [17]. Briefly, the cells were cultured in DMEM supplemented with 10% heat-inactivated fetal bovine serum and 400 μg/ml Geneticin. Cells were grown to approximately 90% confluence in 75 cm^2^culture flasks. Cultures were maintained at 37 °C in a humidified atmosphere containing 5% CO_2_. The medium was changed every 3 days and the cells were passaged approximately once a week by dissociation with a solution of 0.025% trypsin and 0.1% EDTA.

### RNA isolation

Twenty-four hours after treatment of A7r5-α_2B_ cells with 100 nM dexmedetomidine or vehicle, total RNA was isolated using the NucleoSpin® RNA II mini spin kit (Macherey-Nagel, Düren, Germany) (*n* = 3). RNA concentration and purity were confirmed using optical density (OD) measurements at 260 nm and 280 nm (OD_260_/OD_280_ ratio of approximately 2.0).

### DNA microarray gene expression analysis

Microarray experiments were used to determine gene expression profiles in A7r5-α_2B_ cells after treatment with dexmedetomidine or vehicle. Microarray studies were performed at the Finnish DNA Microarray Centre at Turku Centre for Biotechnology. Two hundred nanogram of total RNA from each sample was amplified with Ambion’s Illumina™ RNA TotalPrep Amplification kit (Thermo Fisher Scientific). During the overnight in vitro transcription reaction, cRNA was labeled by biotinylation. Both before and after the amplifications, the RNA/cRNA concentrations were checked with Nanodrop ND-1000 (Thermo Fisher Scientific) and cRNA quality was controlled with the use of BioRad’s (Hercules, CA, USA) Electrophoresis station.

Labelled and amplified material (0.75 μg/array) was hybridized overnight to Illumina’s Sentrix® RatRef-12 BeadChips™ (Illumina Inc., San Diego, CA, USA) at 58 °C according to Illumina® Whole Genome Gene Expression with IntelliHyb Seal protocol (Revision B). Hybridization was detected with 1 μg/ml cyanine3-streptavidine (GE Healthcare Biosciences, Buckinghamshire, UK). The chips were scanned with an Illumina BeadArray™ reader. Numerical results were extracted using Illumina’s BeadStudio™ software without any normalization or background subtraction. The hybridization control report indicated that all hybridizations were successful.

The microarray data were analyzed using R statistical analysis software [23, 24] and the Limma package of the related Bioconductor module [25, 26]. After quality inspection, one outlier sample with considerably lower signal values from the A7r5-α_2B_ control group (vehicle treatment) was discarded. The data were normalized using the quantile normalization method. After statistical testing with Limma, the differentially expressed genes were filtered requiring false discovery rates <0.05 and absolute fold-changes >1.3. This relatively low cut-off in effect size is justified by the exploratory nature of the study and the risk of false negative findings associated with a higher cut-off. Any false positive findings were seen not to cause serious risks for the subsequent pathway analysis and interpretation, as false positives most likely would represent random findings and not results in any systematic bias. The GeneFuncster tool was used to carry out enrichment analysis of all differentially expressed genes towards both Gene Ontology (GO) categories and KEGG pathways [27, 28]. Functional associations of the differentially expressed genes were further analyzed using Ingenuity Pathway Analysis (IPA) software (Ingenuity®Systems [29]).

### Quantitative RT-PCR

To validate the microarray results, 1 μg of the RNA was transcribed to cDNA using the DyNAmo™ cDNA synthesis kit (Thermo Finnzymes, Vantaa, Finland), including controls with no reverse transcriptase enzyme. Quantitative RT-PCR was performed using the SYBR green kit (Kapa Biosystems, Wilmington, MA, USA). Primers specific for the selected genes (Table 1) were designed by using Universal Probe Library Assay Design Center (Roche, Basel, Switzerland). The final concentration of forward and reverse primers in the reaction was 0.2 μM. All measurements were made in triplicate for each sample (*n* = 3). The data were analyzed according to the 2^−ΔΔCt^ method using GAPDH as a reference gene (relative expression to GAPDH) [30].

**Table 1:** Oligonucleotide sequences for quantitative RT-PCR analysis

Gene symbol	Left primer	Right primer
Glrx	GGC TCA GGA GTT TGT GAA CTG CAA G	ATC TGC TTC AGC CGG GCC GT
Cx3cl1	CCA CAA GAT GAC CTC GCC AAT C	TCC ACT GTG GCT GAC TCA GGC T
Cav1	AAC GAC GAC GTG GTC AAG A	CAC AGT GAA GGT GGT GAA GC
Prrx1	CTT CTC CGT CAG TCA CCT GC	CGT GCA AGA TCT TCC CGT AC
GAPDH	CAA CTC CCT CAA GAT TGT CAG CAA	GGC ATG GAC TGT GGT CAT GA

*Glrx* Glutaredoxin, *Cx3cl1* Chemokine (C-X3-C motif) ligand / fractalkine, *Cav1* Caveolin 1, *Prrx1* Paired related homeobox 1, *GAPDH* Glyceraldehyde-3-phosphate dehydrogenase

### Kinase and phosphatase inhibitor screening

#### Compounds

A compound library (former CAT# 2831A) from BioMol (Hamburg, Germany) with 84 known kinase and phosphatase inhibitors (annotations in Additional file 1) was screened. The compounds (and DMSO as control) were plated in 384-well plates at four different concentrations using an automated liquid handling station (Hamilton, Bonaduz, Switzerland). The final concentration range of the compounds was 0.0143 μM, 0.143 μM, 1.43 μM and 14.3 μM. The library screen was performed four times as separate biological replicates.

#### Inhibitor assay

The DELFIA® Cell Proliferation kit (PerkinElmer, Boston, MA, USA), based on the measurement of incorporation of the nucleoside analogue 5-bromo-2′-deoxyuridine (BrdU) during DNA synthesis in proliferating cells, was used to determine the effects of kinase and phosphatase inhibitors on the dexmedetomidine-evoked proliferation response of A7r5-α_2B_ cells. Briefly, A7r5-α_2B_ cells were serum-deprived o/n in DMEM supplemented with 0.5% FBS and seeded into 384-well plates (2.2-2.6 × 10^4^ cells/well) on top of pre-plated inhibitors using a Multidrop™ Combi Reagent Dispenser (Thermo Fischer Scientific, Rockford, IL, USA). Cells were allowed to attach for 2 h at 37 °C before the addition of 100 nM (final concentration) dexmedetomidine or vehicle (DMEM supplemented with 0.5% FBS), each treatment on individual plates. Plates were incubated for 24 h and BrdU (10 μM) was added during the last 4 h. The cells were then fixed and labelled with an anti-BrdU-Eu antibody (0.5 μg/ml) for 75 min at RT under gentle agitation. Cells were washed five times (total 25 min), DELFIA Inducer solution was added and the plates were shaken vigorously for 30 min on a DELFIA plate shaker (PerkinElmer). An EnSight Multimode plate reader (PerkinElmer) was used for signal quantification.

Treatments (dexmedetomidine or vehicle) were performed on separate sample plates and proliferation responses were determined by comparing the inhibitor-treated samples to the DMSO-treated samples (baseline) on each sample plate separately. Total inhibitor effects were determined as an average of four inhibitor concentrations and statistical significance was determined based on these average values.

#### Statistical analysis

For each inhibitor, two-way analysis of variance (ANOVA) was employed to evaluate how concentration and treatment were associated with the proliferation response. All statistical tests were performed as 2-sided, with a significance level set at 0.05. The analyses were performed using SAS System, version 9.3 for Windows (SAS Institute Inc., Cary, NC, USA).

### PamChip® kinase activity profiling

#### Preparation of protein samples for kinase activity profiling

A7r5-α_2B_ cells were plated in 60 mm dishes and grown to approximately 90% confluence followed by serum deprivation o/n in DMEM supplemented with 0.5% FBS. Two series of dishes were treated in parallel with 100 nM dexmedetomidine (or vehicle) by replacing the entire medium for 5 min, 30 min, 2 h or 24 h. For each time point, 2 samples were treated with dexmedetomidine and 2 samples served as controls. After exposure for the desired time, the dexmedetomidine (or vehicle) solution was aspirated from the first series of samples, then the dishes were placed on ice and the cells were washed twice with ice-cold PBS. Cells were lysed with ice-cold M-PER Mammalian Extraction Buffer (Thermo Fischer Scientific) containing Halt™ phosphatase (1/100) and protease inhibitors (1/100) (both from Thermo Fischer Scientific). Lysates were incubated for 15 min in a shaking ice bath. Cell lysis was confirmed visually and completed by scraping. The lysates from the first series were transferred to the replicate dishes so as to lyse the contents of both dishes in the same buffer. Cell lysates were centrifuged for 15 min at 16.000 x g at 4 °C and supernatants were collected into clean vials, snap-frozen with liquid nitrogen and stored at −70 °C. Protein concentrations were determined with a protein assay kit (Pierce™ BCA protein assay kit, Thermo Fischer Scientific).

#### Protein kinase activity profiling

Kinase activity profiles were determined using the PamChip® 12 serine/threonine (STK) and protein tyrosine (PTK) peptide microarray system (PamGene International B.V., ‘s-Hertogenbosch, The Netherlands) [31–34].

To prevent non-specific binding, the arrays on the PamChip® 12 STK chips were incubated with 2% bovine serum albumin (BSA) in water for 30 cycles (15 min). Arrays were then washed three times with kinase assay buffer (50 mM Tris-HCl pH 7.5, 10 mM MgCl_2_, 1 mM EGTA, 2 mM DTT, 0.01% Brij35). Reaction mixtures contained 0.01% BSA in kinase assay buffer supplemented with anti-phospho-Ser/Thr antibodies (PamGene International BV [31],) in a final volume of 40 μl per array. For each STK assay, 0.5 μg of sample protein was present in the reaction mixture. The reaction was initiated by the addition of ATP (final concentration 400 μM). Samples were pumped up and down through the porous membrane of the arrays for 60 cycles (in total 60 min). Arrays were washed and then incubated with a secondary antibody (polyclonal swine anti-rabbit Immunoglobulin/FITC) for 30 min. Images (10, 50 and 200 msec exposure time) were captured every 5 min with an integrated CCD-based optical system combined with Evolve software (version 1.5, PamGene International BV). The secondary antibody was removed and arrays were washed before post-wash images were taken at different exposure times (20, 50, 100 and 200 msec).

The PTK assay mixture contained kinase assay buffer, 0.01% BSA and 400 μM ATP, supplemented with 4 μl protein kinase-additive (PamGene International BV), 10 mM DTT and FITC-labeled anti-phosphotyrosine antibody (PamGene International BV). For each PTK assay, 5 μg of sample protein was used. Since a labeled antibody is present in the PTK assay mixture, peptide phosphorylation was monitored during incubation with assay mixture by capturing images every 5 min (10, 20, 50 and 200 msec exposure time), allowing real time recording of the reaction kinetics (one-step reaction). Arrays were washed and fluorescence was detected at different exposure times (20, 50, 100 and 200 msec).

#### Signal quantification

Fluorescence signal intensities for all peptides were analyzed using BioNavigator 6.1 software (PamGene International BV [35]), a statistical analysis and visualization software tool. Around each spot a local background was calculated, and then this value was subtracted from the signal intensity, resulting in SigmBg. For signal quantification, the slope of the SigmBg versus exposure times was calculated to increase the dynamic range. Visual quality control was done to exclude defective arrays from the analysis. Peptides with CV < 30% for the replicates were included in the analysis, resulting in 96 peptides for PTK and 98 peptides for STK analysis.

#### Statistical analysis

Significant effects (*p* < 0.05) were identified by fitting a model for the conditions which performs a Dunnet’s test for multiple conditions against a single control.

#### Pathway analysis and upstream kinase analysis

Peptides found to be significantly differently phosphorylated between dexmedetomidine-treated samples and their vehicle-treated controls (*t* = 30 min and *t* = 24 h) were used for pathway analysis with the canonical pathway analysis program MetaCore™ (Thomson Reuters, St. Joseph, MI, USA). The top 10 most significant process networks were identified and relevant signaling networks were assembled based on manually curated objects generated by log fold-change data. Pathways were ranked by -log (p), and -log (p) > 4 were considered significant. BioNavigator software was used to perform upstream PTK and STK analysis by comparing differentially phosphorylated peptides between dexmedetomidine-treated samples and their vehicle-treated controls (*t* = 30 min and *t* = 24 h) and linking (derived from the Kinexus phosphoNET database [36]) them to the putative upstream kinases responsible for their phosphorylation. The upstream kinase analysis tool generated hypotheses about kinases that were differentially active between dexmedetomidine-treated samples and their vehicle-treated controls.

## Results

### Illumina DNA microarray

**Fig. 1:**
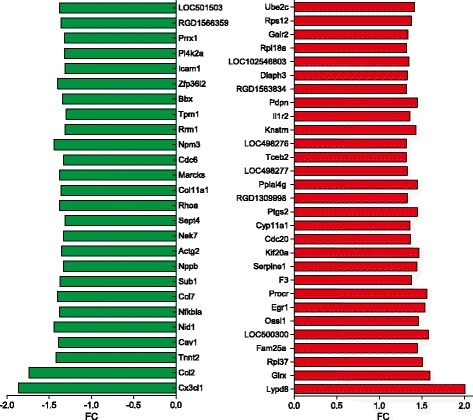
Fold change (FC) values of upregulated (*red*) and downregulated (*green*) genes in dexmedetomidine-treated (100 nM) A7r5-α_2B_ vascular smooth muscle cells compared with vehicle-treated control cells in the Illumina DNA microarray. The genes are ordered based on the associated adjusted *p-*values, in decreasing order. Explanation of gene symbols, actual and adjusted *p-*values can be found in Additional file 2

The objective of this set of experiments was to identify specific genes involved in α_2B_-adrenoceptor-evoked VSMC proliferation. To define the effects of activated α_2B_-adrenoceptors on gene expression profiles, A7r5-α_2B_ VSMCs were incubated with 100 nM dexmedetomidine for 24 h. Of the 22,000 genes (22,523 transcripts) analyzed, 55 genes (see Additional file 2) were differentially expressed in dexmedetomidine-treated cells compared with vehicle-treated controls (FC > 1.3 and *p* < 0.05; *p*-values corrected for false positive discovery rates). Out of these genes, 29 were upregulated with fold change (FC) values ranging from 1.32 to 2.01 and 26 were downregulated with FC values ranging from −1.3 to −1.86 (Fig. 1). Most of the genes identified have not been reported previously to be differentially expressed after dexmedetomidine treatment. To evaluate the functional associations of the observed gene expression profiles, the most significantly enriched biological processes, cellular components and functions among differentially expressed genes were identified.

#### GeneFuncster functional enrichment analysis

**Fig. 2:**
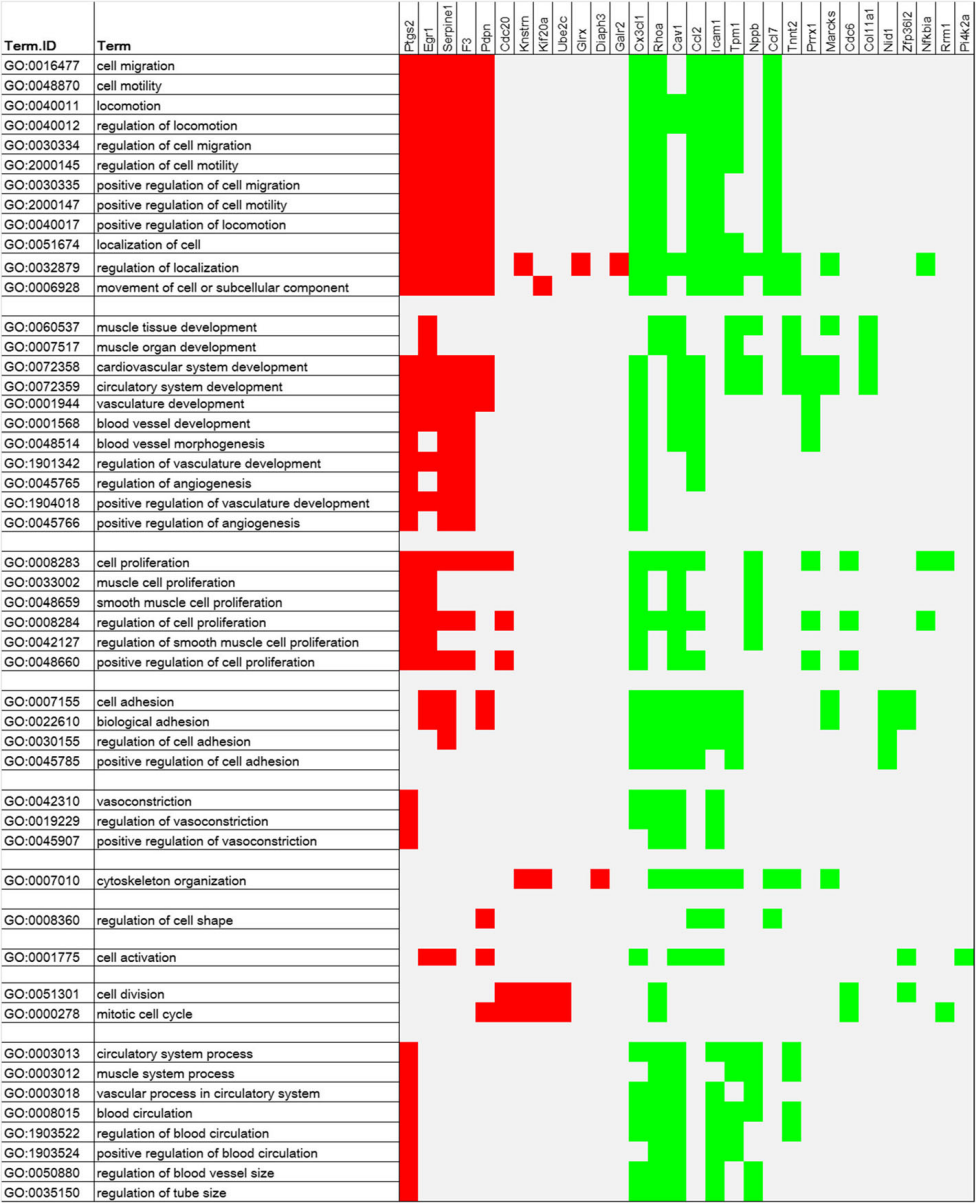
Selected significantly enriched (*p* < 0.01) biological process GO terms related to cardiovascular functions in upregulated (*red*) and downregulated (*green*) genes induced by α_2B_-adrenoceptor activation upon dexmedetomidine stimulation in A7r5-α_2B_ vascular smooth muscle cells. Explanation of gene symbols in Additional file 2

We analyzed the up- and downregulated gene lists by using the GeneFuncster tool, which can analyze the functional enrichment in short filtered gene lists towards both GO and KEGG and provide comprehensive result visualization for both databases (*p*-value limit for terms with detailed results: 0.01) [27]. In general, a high number (> 200) of significantly (*p* < 0.01) enriched biological processes was found among the differentially expressed genes induced by α_2B_-adrenoceptor activation. Analysis of cellular component GO terms and molecular function GO terms resulted in 29 and 10 significant enrichments, respectively (Additional file 3). Of the first 200 enriched biological process GO terms, 49 were related to cardiovascular system development, blood circulation, cell migration and motility, cell proliferation, cell adhesion, vasoconstriction, mitotic cell cycle, cytoskeleton organization and regulation of cell shape (Fig. 2). Notably, GO terms such as “regulation of smooth muscle cell proliferation” (GO: 0042127), “positive regulation of cell proliferation” (GO: 0048660) and “positive regulation of vasculature development” (GO: 1,904,018) were among the enriched biological process GO terms.

Some of the differentially expressed genes were involved in several aspects of cell function, including cardiovascular system development, regulation of proliferation, migration and adhesion, vasoconstriction and mitotic cell cycle. Upregulated genes which had the largest number of annotations within the selected 49 biological process GO terms were prostaglandin-endoperoxide synthase 2 (Ptgs2 or COX2; 38 annotations), early growth response 1 (Egr1; 29 annotations), serpin peptidase inhibitor (Serpine1 or plasminogen activator inhibitor type 1; PAI-1; 28 annotations), coagulation factor III, tissue factor (F3; 24 annotations), and podoplanin (Pdpn; 21 annotations). Downregulated genes with the most annotations per gene were chemokine (C-X3-C motif) ligand 1 (Cx3cl1 or fractalkine; 40 annotations), Rhoa (34 annotations), Cav1 (33 annotations), chemokine (C-C motif) ligand 2 (ccl2; 29 annotations), Icam1 (28 annotations) and tropomyosin 1 alpha (Tpm1; 23 annotations). Among the differentially expressed genes were many genes that are known to participate in the regulation of proliferation in different types of cells, such as Egr1, Serpine1, Pdpn, Cx3cl1, glutaredoxin (Glrx), cell division cycle 20 (cdc20), kinesin family member 20a (Kif20a) and ubiquitin-conjugating enzyme E2C (ube2c). However, in A7r5 VSMCs, very little is known about the function of most of these genes.

#### Ingenuity® pathway analysis

The results of the Illumina DNA microarray were imported into the Ingenuity® pathway analysis (IPA) application and a functional pathway analysis was performed to identify significant biological functions, networks and upstream regulators related to the differentially expressed genes in dexmedetomidine-stimulated vs. vehicle-treated A7r5-α_2B_ cells. The top 10 up- and down-regulated genes identified by IPA Core Functional Analysis are listed in Table 2 and include partly the same genes that were determined to be involved in many of the functionally enriched biological process GO terms in the GeneFuncster functional enrichment analysis: Egr1, Glrx, Kif20a, Cx3cl1, Rhoa, Cav1 and Ccl2.

**Table 2:** Differentially expressed genes following α_2B_-adrenoceptor activation compared with vehicle-treated control according to IPA

Gene symbol	Gene name	Fold change	*P*-value
Up-regulated genes			
Lypd8	LY6/PLAUR domain containing 8	2.01	4.2·10^−6^
Glrx	glutaredoxin	1.60	1.0·10^−3^
LOC500300	similar to hypothetical protein MGC6835 (Depp)	1.58	4.0·10^−3^
Procr	protein C receptor	1.56	5.7·10^−3^
Egr1	early growth response 1	1.54	5.0·10^−3^
Rpl37	ribosomal protein L37	1.51	1.0·10^−3^
Kif20a	kinesin family member 20a	1.47	1.3·10^−2^
Oasl	2′-5′-oligoadenylate synthetase-like	1.46	5.0·10^−3^
Fam25a	family with sequence similarity 25, member A	1.45	4.0·10^−3^
Ppial4g	peptidylprolyl isomerase A like 4G	1.45	2.3·10^−2^
Down-regulated genes			
Cx3cl1	chemokine (C-X3-C motif) ligand 1	−1.86	1.7·10^−5^
Ccl2	chemokine (C-C motif) ligand 2	−1.74	4.4·10^−4^
Ahnak	AHNAK nucleoprotein	−1.47	2.3·10^−2^
Npm3	nucleophosmin/nucleoplasmin 3	−1.45	3.0·10^−2^
Nid1	nidogen 1	−1.44	8.7·10^−3^
Tnnt2	troponin T2, cardiac type	−1.42	6.0·10^−3^
Zfp36l2	zinc finger protein 36, C3H type-like 2	−1.40	4.4·10^−2^
Ccl7	chemokine (C-C motif) ligand 7	−1.40	2.0·10^−2^
Cav1	caveolin 1	−1.39	8.2·10^−3^
Rhoa	ras homolog family member A	−1.38	2.4·10^−2^

The most significant biological functions predicted by the IPA core analysis included (changes in) cellular movement, cell-to-cell signaling, cellular growth and proliferation, cellular development, skeletal and muscular system development and function and cell cycle, with a total of 42 putative genes (Table 3A). Moreover, these categories contained the following functions specifically related to smooth muscle cells (SMCs): migration (5 genes), adhesion (2 genes), proliferation (9 genes) and contraction (3 genes). A further gene set with 15 altered gene targets was involved in cell cycle regulation. *P*-values in the range of 1.34 × 10^−10^ to 2.49 × 10^−3^ indicated statistical significance. Table 3B summarizes the differentially expressed genes involved in SMC-specific functions and includes the upregulated genes Egr1, Ptgs2, Serpine1 and F3 and the downregulated genes Rhoa, Cx3cl1, Cav1, natriuretic peptide b (Nppb or BNP) and paired related homeobox 1 (Prrx1). Egr1, Ptgs2, Serpine1 and F3 were involved in migration and proliferation of SMCs. Ptgs2 was also involved in SMC contraction. Downregulation of Rhoa was associated with migration, adhesion, proliferation and contraction of SMCs. Downregulation of Cx3cl1 was associated with adhesion and proliferation. Downregulation of Cav1 was associated with VSMC proliferation and contraction. Other downregulated genes associated with cell proliferation were Nppb and Prrx1. The full list of significant biological functions and diseases identified by IPA can be found in Additional file 4.

**Table 3:** Significant biological functions predicted by IPA (A) and differentially expressed genes involved in SMC-specific functions (B)

A. Significant biological functions (42 genes in total)		
Category	*P*-value	Number of involved genes
Cellular movement	1.34∙10^−10^ − 2.31∙10^−3^	25
Migration of SMCs	9.87∙10^−5^	5
Migration of VSMCs	2.35∙10^−4^	4
Cell−to−cell signaling and interaction	1.34∙10^−10^ − 2.45∙10^−3^	19
Adhesion of SMCs	7.95∙10^−4^	2
Cellular growth and proliferation	4.21∙10^−7^ − 2.49∙10^−3^	27
Proliferation of SMCs	4.21∙10^−7^	9
Skeletal and muscular system development and function	4.21∙10^−7^ − 2.24∙10^−3^	18
Contraction of SMCs	2.17∙10^−3^	3
Cell cycle	5.27∙10^−7^ − 1.50∙10^−3^	15
B. Differentially expressed genes involved in SMC−specific functions		
Category	Involved genes	
Migration of SMCs	↑ Egr1, ↑ Ptgs2, ↑ Serpine1, ↑ F3, ↓ Rhoa	
Migration of VSMCs	↑ Egr1, ↑ Ptgs2, ↑ Serpine1, ↓ Rhoa	
Adhesion of SMCs	↓ Cx3cl1, ↓ Rhoa	
Proliferation of SMCs	↑ Egr1, ↑ Ptgs2, ↑ Serpine1, ↑ F3, ↓ Nppb, ↓ Cx3cl1, ↓ Rhoa, ↓ Prrx1, ↓ Cav1	
Contraction of SMCs	↑ Ptgs2, ↓ Rhoa, ↓ Cav1	

↑ = upregulated, ↓ = downregulated, Egr1 = early growth response 1, Ptgs2 = prostaglandin−endoperoxide synthase 2 (COX−2), Serpine1 = serpin family E member 1 (plasminogen activator inhibitor−1 = PAI−1), F3 = coagulation factor III, tissue factor, Rhoa = ras homolog family member A, Cx3cl1 = chemokine (C−X3−C motif) ligand 1, Nppb = natriuretic peptide B, Prrx1 = paired related homeobox 1, Cav1 = caveolin 1

#### Comparison of genes identified with GeneFuncster and IPA

**Fig. 3:**
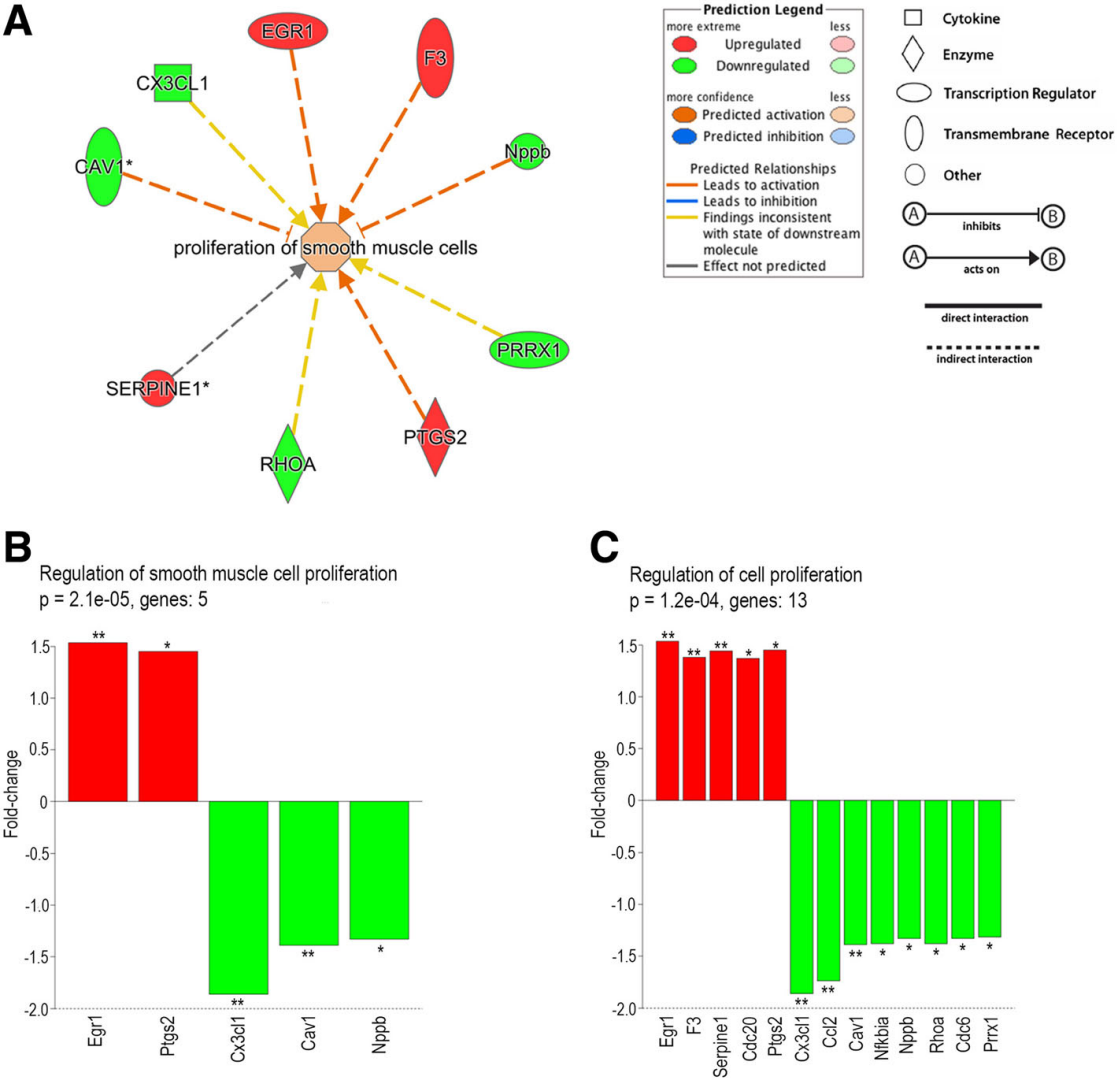
Network of differentially expressed genes in dexmedetomidine-stimulated A7r5-α_2B_ vascular smooth muscle cells involved in the regulation of smooth muscle cell proliferation according to Ingenuity Pathway Analysis (**a**) and differentially expressed genes annotated in the biological process GO terms “regulation of smooth muscle cell proliferation” (GO:0042127) (**b**) and “positive regulation of cell proliferation” (GO:0048660) (**c**) according to GeneFuncster functional enrichment analysis. *Red* and *green* colors indicate upregulation and downregulation, respectively. Explanation of gene symbols in Additional file 2

We have shown in our earlier study that proliferation of A7r5-α_2B_ VSMCs is significantly increased when the cells are stimulated with the α_2_-adrenoceptor agonist dexmedetomidine [17]. In the current study, the expression of a number of genes involved in VSMC proliferation was changed as a consequence of α_2B_-adrenoceptor activation in dexmedetomidine-treated A7r5-α_2B_ cells as identified by IPA core analysis (Fig. 3a) and GeneFuncster functional enrichment analysis (Fig. 3b and c). According to the IPA core analysis, upregulation of Egr1 (FC 1.54; *P* = 0.005), F3 (FC 1.38; *P* = 0.008) and Ptgs2 (FC 1.45; *P* = 0.02) is predicted to lead to activation of SMC proliferation. Cav1 (FC -1.39; *P* = 0.008) and Nppb (FC -1.33; *P* = 0.02) are predicted to have inhibitory effects on SMC proliferation, which is in line with the observed decreased expression of these genes. According to IPA, Cx3cl1, Prrx1 and Rhoa have indirect activating effects on SMC proliferation. However, in our results, Cx3cl1 (FC -1.86; *P* = 0.00002), Prrx1 (FC -1.32; *P* = 0.04) and Rhoa (FC -1.38; *P* = 0.02) were downregulated, which is inconsistent with the predicted actions of these genes in the IPA core analysis. Serpine1 (FC 1.44; *P* = 0.008) is indicated to be involved in the proliferation of SMCs, but IPA does not predict the direction of the effect. GeneFuncster identified the biological process GO terms “regulation of smooth muscle cell proliferation” and “regulation of cell proliferation” as significantly enriched, with *P*-values of 2.1 × 10^−5^ and 1.2 × 10^−4^, respectively. The differentially expressed genes linked to these GO terms were mainly the same as the ones identified by IPA. Egr1, Ptgs2, Cx3cl1, Cav1 and Nppb were linked to both GO terms (Fig. 3b and c). In GeneFuncster, F3, Serpine1, Rhoa and Prrx1 were linked only to general regulation of cell proliferation, although IPA identified them as genes regulating SMC proliferation.

#### IPA upstream regulator analysis

**Fig. 4:**
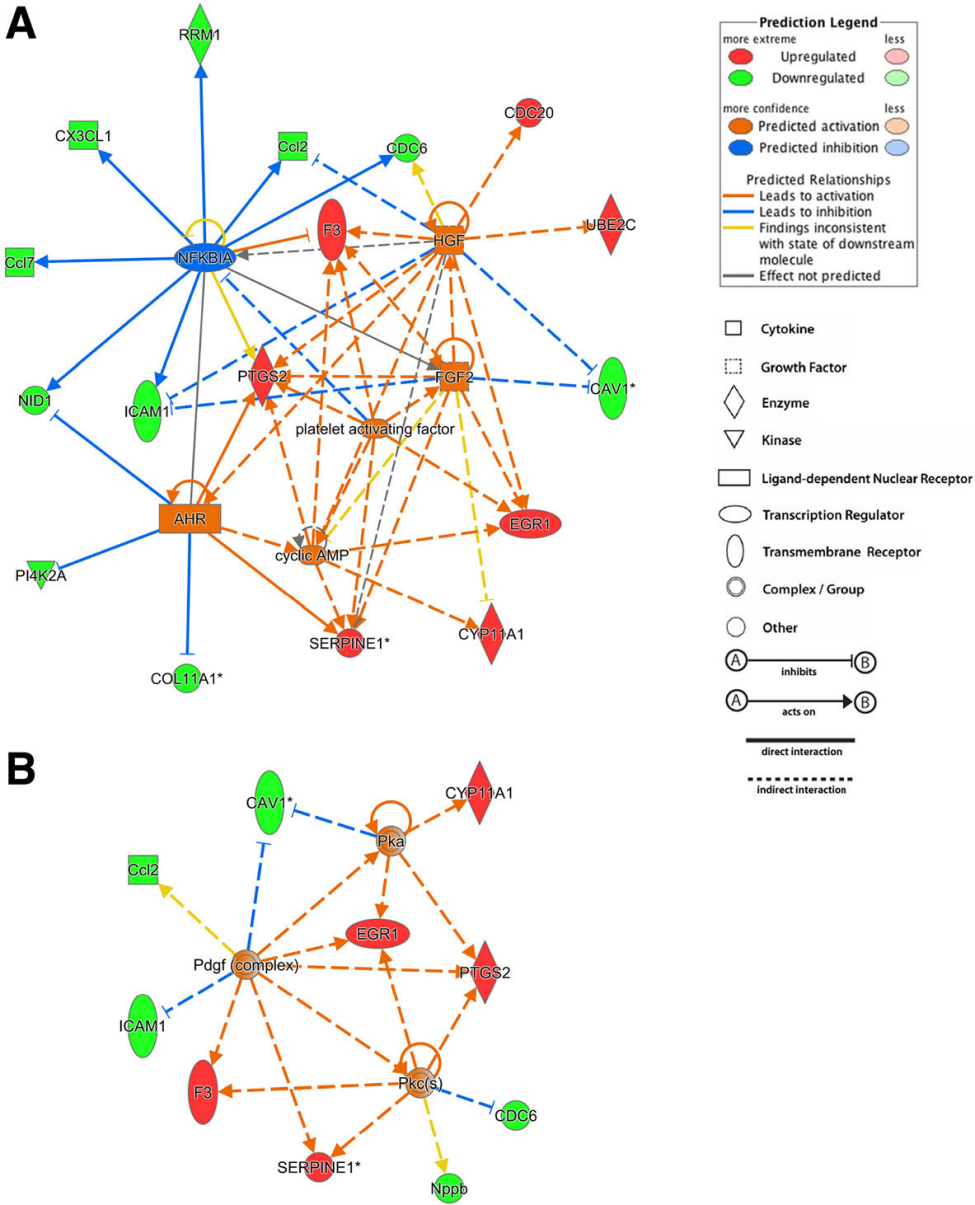
Upstream regulators identified by Ingenuity Pathway Analysis, predicted to participate in the regulation of gene expression upon α_2B_-adrenoceptor activation in A7r5-α_2B_ vascular smooth muscle cells. The network of upstream regulators with │z-score│ > 2 is illustrated in panel (**a**). The network of PKC, PKA and Pdgf targets is presented in panel (**b**). *Red* and *green* colors indicate upregulation and downregulation, respectively. Explanation of gene symbols in Additional file 2

We also conducted upstream regulator analysis using IPA. This analysis determines likely upstream regulators that are connected to dataset genes through a set of direct or indirect relationships and predicts their activation state. For a particular regulator, the overlap *P*-value measures enrichment of genes regulated by this regulator in the dataset without taking into account the regulation direction. The activation z-score is used to predict activation or inhibition of regulators based on relationships with dataset genes and direction of change of dataset genes and a prediction of effect on the function, increased or decreased, is given for |z-score| >2. For our dataset, upstream regulator analysis identified six upstream regulators with |z-score| >2: platelet activating factor (PAF), hepatocyte growth factor (HGF), basic fibroblast growth factor (bFGF/FGF2), cyclic AMP (cAMP) and aryl hydrocarbon receptor (AHR) were predicted to be activated and NF-kappa-B inhibitor alpha (NFKBIA) was predicted to be inhibited. The predicted effects of these upstream regulators on differentially expressed genes in our dataset are illustrated in Fig. 4a. There were 9 genes (proteins) in the dataset showing regulatory effects by NFKBIA. Among them, 8 genes had expression change directions consistent with the inhibition of NFKBIA (z-score = −2.482, overlap *P*-value = 1.9 × 10^−7^). Seven of nine genes and 10 of 11 genes showed expression change directions consistent with the activation of FGF2 (z-score = 2.140, overlap *P*-value = 3.6∙10^−5^) and HGF (z-score = 2.203, overlap *P*-value = 4.9∙10^−7^), respectively. Five genes indicated regulatory effects by cAMP (z-score = 2.141, overlap *P*-value = 5.1∙10^−4^), six genes by PAF (z-score = 2.159, overlap *P*-value = 1.4∙10^−7^) and seven genes by AHR (z-score = 2.183, overlap *P*-value = 1.5∙10^−3^), and all genes had expression change directions consistent with the activation of these upstream regulators.

Protein kinase C (different isoforms, PKCs), protein kinase A (PKA) and platelet derived growth factor (PDGF) obtained z-scores of 1.788, 1.980 and 1.988, respectively. Because they did not reach a z-score of 2, IPA did not give a prediction on their activation state. However, based on their relatively high z-scores, activation may be assumed. PKC, PKA and PDGF are known to regulate the proliferation of many cell types, including VSMCs; therefore, we inspected their roles as upstream regulators of the genes in our dataset (Fig. 4b). Six genes demonstrated regulatory effects by PKCs and among them, five genes had expression change directions consistent with the assumed activation of PKCs (overlap *P*-value = 1.7∙10^−5^). Six of seven genes and four of four genes showed changes consistent with the assumed activation of Pdgf (overlap *P*-value = 6.0∙10^−9^) and PKA (overlap *P*-value = 3.9∙10^−4^), respectively.

#### Validation by RT-PCR

To validate the microarray results, we analyzed the expression of selected genes by means of quantitative RT-PCR, using the same samples as in the microarray experiment. These genes were chosen to represent a spectrum of significant expression level changes in the microarray. Out of the differentially expressed genes that were annotated within the selected 49 biological process GO terms, Glrx was the most upregulated gene (FC 1.60) and Cx3cl1 was the most downregulated gene (FC −1.86). Cav1 (FC −1.39) and Prrx1 (FC −1.32) represent genes with smaller expression level changes. All selected genes were associated with significantly enriched biological process GO terms or the network of differentially expressed genes involved in the regulation of SMC proliferation shown in Figs. 2 and 3, respectively. Consistent with the microarray results, the expression differences (FC) for Glrx and Cx3cl1 were 1.75 and −3.02, respectively. Cav1 and Prrx1 represented genes with smaller FC-values in the microarray; they did not show differential expression compared to control samples in the RT-PCR experiments (Table 4).

**Table 4:** RT-PCR verification of selected differentially expressed genes in the dexmedetomidine-treated vs. vehicle-treated control samples

Gene symbol	Gene name	Microarray *p*-value	Microarray FC	RT-PCR FC
Glrx	glutaredoxin 1	0.0010	1.60	1.75
Cx3cl1	chemokine (C-X3-C motif) ligand 1	0.000017	−1.86	−3.02
Cav1	caveolin 1	0.0082	−1.39	−1.05
Prrx1	paired related homeobox 1	0.045	−1.32	−1.01

### Screening of a kinase/phosphatase inhibitor library

The objective of this set of experiments was to investigate the effects of different kinase and phosphatase inhibitors on the α_2B_-adrenoceptor-evoked proliferation in A7r5-α_2B_ VSMCs and possibly identify specific signaling pathways involved in the proliferation response. We screened an 84-compound library consisting of 70 kinase inhibitors and 14 phosphatase inhibitors for their effects on cell proliferation.

**Fig. 5:**
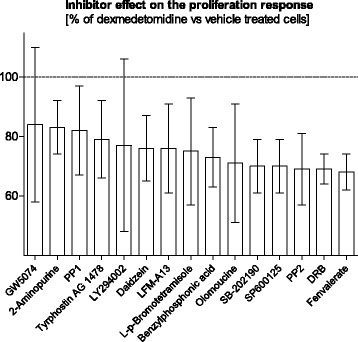
Effects of statistically significant (*p* < 0.01) inhibitors on the proliferation of dexmedetomidine-stimulated A7r5-α_2B_ vascular smooth muscle cells, in relation to vehicle-treated control cells. Treatments (dexmedetomidine or vehicle) were performed on separate sample plates and proliferation responses were determined by comparing the inhibitor-treated samples to the DMSO-treated samples (baseline) on each sample plate separately. Total inhibitor effects were determined as an average of four inhibitor concentrations and statistical significance was determined based on these average values. Error bars indicate standard deviation

The inhibitor responses showed rather large variation between the biological replicates, and statistical analysis was employed to evaluate the significance of the inhibitor effects. We detected altogether 15 compounds that inhibited the dexmedetomidine-induced proliferation response in A7r5-α_2B_ cells with statistical significance (*p* < 0.01). Another 18 inhibitors were associated with trend-level effects with 0.01 < *p* < 0.05. Statistical significance of inhibitor effects was determined by comparing averaged inhibitor effects (average of four concentrations) on the proliferation response in dexmedetomidine- and vehicle-treated cells. Statistically significant (*p* < 0.01) inhibitors repressed the dexmedetomidine-induced proliferation response by 16 to 32% when compared to vehicle-treated control cells (Fig. 5). Their potential target kinases and phosphatases are listed in Table 5, and include receptor (EGFR) and cytosolic (Src, BTK) tyrosine kinases, serine/threonine kinases (p38 MAPK, CK2, JNK, Cdks, Raf-1) as well as phosphatases (calcineurin, protein tyrosine phosphatases) known for their important functions in cell signaling and regulation of cell proliferation, differentiation, migration and apoptosis. More information on the pathways possibly involved in the observed effects is given in Additional file 5.

**Table 5:** Significant (*p* < 0.01) inhibitors, their potential target kinases and phosphatases and their effects on VSMC proliferation

Inhibitor	Inhibition of proliferation [% of control]	Potential target kinases and phosphatases	Effect of target kinase/phosphatase on VSMC proliferation	Reference
Fenvalerate	32	calcineurin	↑	[61, 173–175]
5,6-dichloro-1-beta-D-ribofuranosyl benzimidazole	31	CK2	↑	[176, 192, 303–305]
		Cdk9	↑	[306, 307]
PP2	31	Src family kinases	↑	[177–182, 308]
PP1	18			
SP600125	30	JNKs	↑	[183, 184]
SB-202190	30	p38 MAPKs	↑	[185–187]
Olomoucine	29	Cdk1, Cdk2	↑	[85, 188–191]
Benzylphosphonic acid	27	tyrosine phosphatases	↑/↓	[309–311]
L-p-bromotetramisole	25	alkaline phosphatase	n.d.	[312, 313]
		tyrosine phosphatases	↑/↓	[309–311]
LFM-A13	24	BTK, Plk, Jak2	↑	[314–317]
Daidzein	24	*arrests cell cycle at G1*	↓	[318–320]
LY294002	23	PI3-kinases	↑	[173, 180, 192–195]
Tyrphostin AG 1478	21	EGFR	↑	[179, 196–199]
2-Aminopurine	17	PKR	↓	[321, 322]
GW5074	16	Raf-1	↑	[37, 177, 200]

CK2 = protein kinase CK2 (casein kinase), Cdks 1,2 and 9 = cyclin-dependent kinases 1,2 and 9, Src family kinases = Src, Fyn, Hck and Lck, JNKs = c-Jun N-terminal kinases, p38 MAPKs = p38 mitogen-activated protein kinases, BTK = Bruton’s tyrosine kinase, Plk = Polo-like kinase, Jak2 = Janus kinase 2, PI3 kinase = phosphoinositide 3-kinase, EGFR = epidermal growth factor receptor, PKR = ds-RNA-activated protein kinase, Raf-1 = RAF proto-oncogene serine/threonine-protein kinase. In cases when little data are available in VSMCs, the table was augmented with information of kinase/phosphatase effects on cell proliferation in other cell types

### Kinase activity profiling

#### PTK and STK activity profiles of dexmedetomidine-stimulated A7r5-α_2B_ cells

The objective of this set of experiments was to investigate the activity profiles of protein tyrosine kinases (PTK) and serine/threonine kinases (STK) in A7r5-α_2B_ cells after 5 min, 30 min, 2 h or 24 h stimulation with the agonist dexmedetomidine, and to further identify signaling proteins/pathways involved in the generation of the α_2B_-adrenoceptor-evoked proliferation response. We searched for new targets of α_2B_-adrenoceptor signaling using two different microarrays with 144 peptides on each chip, representing known phosphorylation sites of PTKs or STKs. PTK and STK profiling of dexmedetomidine- vs. vehicle-treated A7r5-α_2B_ cells resulted in clear activity signals and high experimental quality. Out of the 144 peptides, 96 peptides on the PTK chip and 98 peptides on the STK chip were detected above threshold level in one or more of the samples.

**Fig. 6:**
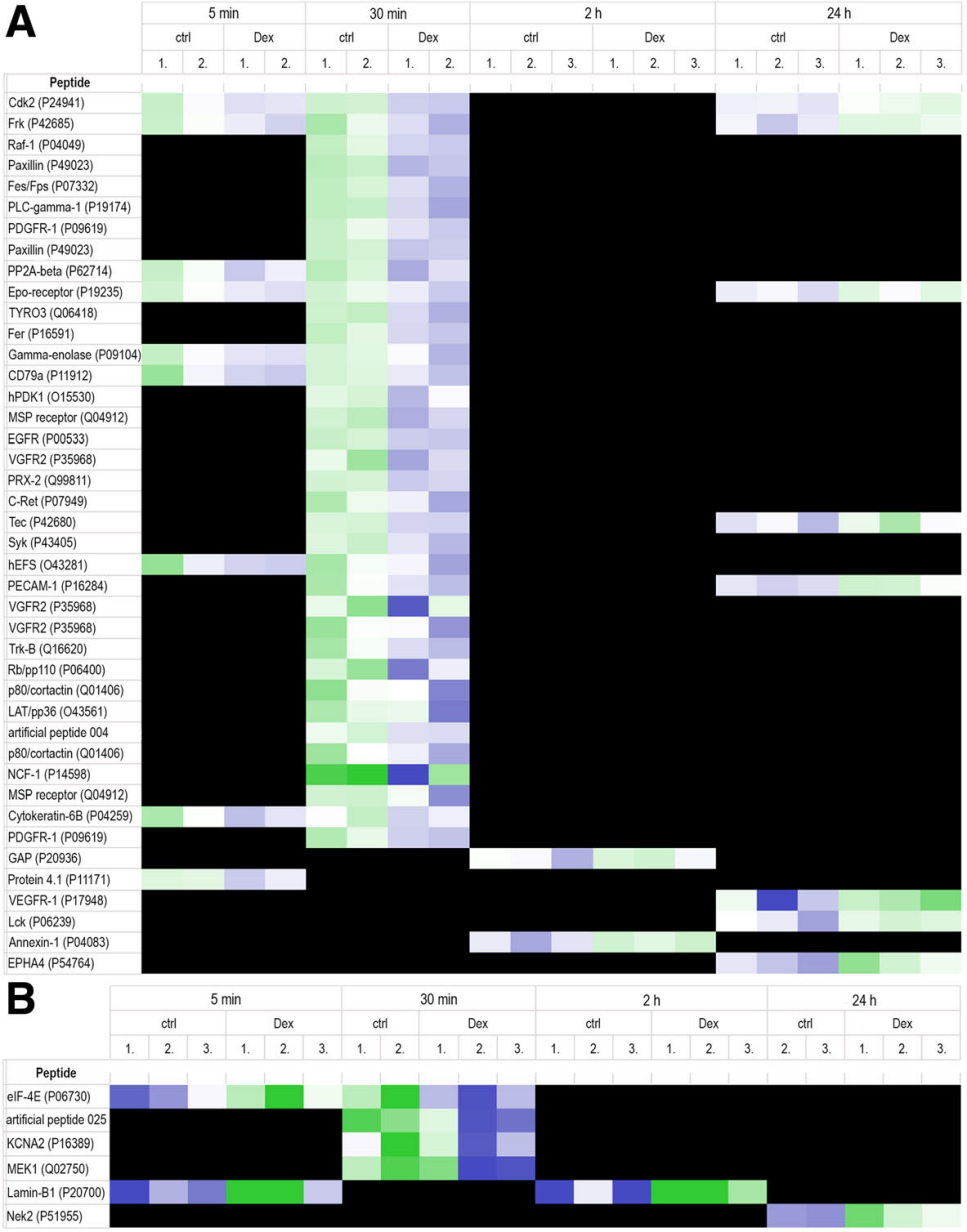
Protein tyrosine kinase (PTK, panel **a**) and serine/threonine kinase (STK, panel **b**) activity profiles at 5 min, 30 min, 2 h and 24 h in A7r5-α_2B_vascular smooth muscle cells treated with 100 nM dexmedetomidine (DEX) or vehicle (ctrl). Peptides on the chips represent known phosphorylation sites of PTKs and STKs; for clarity, the names of these kinases and their UniProt identifiers are used instead of the peptide sequences. Two artificial (ART025 and ART004) peptides are included in the figure; they are not derived from natural proteins, but they were selected from a library of peptides known to be targets of kinase phosphorylation. Green and *blue* colors indicate increased or decreased kinase activity, respectively, in dexmedetomidine-treated cells when compared with vehicle-treated controls. *Black* color indicates no differences between dexmedetomidine-treated cells and vehicle-treated controls. The replication CV of high signal spots on the PTK and STK chips was <20%, indicating good experimental quality. *n* = 3 per time point; except for *t* = 5 min in the PTK experiment, *t* = 30 min (ctrl) and *t* = 24 h (ctrl) in the STK experiment, where *n* = 2

**Fig. 7:**
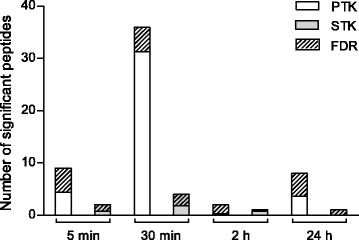
Number of peptides with significantly increased or decreased phosphorylation (*p* < 0.05) and expected proportion of false discoveries (FDR) on protein tyrosine kinase (PTK) and serine/threonine kinase (STK) chips at different time points. The FDR bar indicates the expected proportion (%) of false discoveries per time point on each chip

Figure 6 shows statistically significant (*p* < 0.05) effects of dexmedetomidine exposure at different time points on the PTK (A) and STK (B) chips, being either significantly increased (green) or decreased (blue) compared with vehicle-treated control samples. Most remarkable differences in kinase activation profiles were observed after 30 min dexmedetomidine exposure, where altogether 40 peptides (PTK + STK) showed decreased phosphorylation when compared to vehicle-treated controls (Fig. 7). The generated tyrosine kinase and serine/threonine kinase activity profiles showed that dexmedetomidine stimulation induced transient decreases of kinase signaling at the early time points, 5 min and 30 min, which then recovered at the later time points resulting in slight activation of kinase signaling at 24 h. Changes in kinase activities were more substantial on the PTK chip: decreased phosphorylation of 9 peptides or 36 peptides was detected after 5 min or 30 min of dexmedetomidine exposure, respectively. On the STK chip, increased or decreased phosphorylation was detected in 2 (5 min) and 4 peptides (30 min), respectively (Fig. 7). After 24 h of dexmedetomidine exposure, slightly increased kinase activities could be detected; increased phosphorylation of 8 peptides on the PTK chip and 1 peptide on the STK chip was seen.

#### Canonical pathway analysis of kinase activity data significantly changed at 30 min and 24 h

At the 30 min time point, significant decreases of Raf-1, MEK1 and MEK2 phosphorylation were detected following dexmedetomidine-induced α_2B_-adrenoceptor activation. The Raf-1-MEK-ERK pathway is one of the best characterized MAP kinase signaling pathways, known to regulate cell proliferation [37]. Also phosphorylation of epidermal growth factor receptor (EGFR), known to provoke the activation of MAP kinases, was significantly decreased. In addition, decreased phosphorylation of linker for activated T cells (LAT), platelet derived growth factor receptor (PDGFR), erythropoietin receptor (Epo-receptor), spleen tyrosine kinase (Syk), phospholipase C γ (PLCγ), phosphoinositide-dependent kinase (PDK) and tyrosine protein kinase Fer were detected.

The kinase phosphorylation activities measured on peptides showing different responses on both PTK and STK chips were further evaluated by canonical pathway analysis (GeneGo MetaCore™) at 30 min, where kinase signaling was clearly decreased, and at 24 h, where kinase signaling was reversed to slight activation. Pathways with -log(p) > 4 were considered significant. At the 30 min time point, more than 10 pathways were identified as significant with –log(p) ranging from 7 to 10 (Additional file 6); dexmedetomidine-induced α_2B_-adrenoceptor activation inhibited all of these pathways. Based on their names, most of the significant pathways appeared less relevant from the point of view of α_2B_-adrenoceptor-evoked cell proliferation; nonetheless, also the following pathways containing pertinent signaling cascades were included in the top ten significant pathways: “Signal transduction_IP3 signaling”, “Development of EPO-induced PI3K/AKT pathway and Ca^2+^ influx” and “Development of EPO-induced MAPK pathway”. The Raf-1 - MEK1 - MEK2 signaling cascade appeared in most of these pathways, as well as interactions of PLCγ with LAT, PDGFR or Syk and interactions of the Epo-receptor with Syk and PLCγ. In addition, EGFR, PDK and Fer appeared in many of the significant pathways.

At the 24 h time point, increased phosphorylation by altogether 9 kinases was observed (Fig. 6), indicating delayed α_2B_-adrenoceptor-evoked increases in kinase activity. However, all pathways identified by the canonical pathway analysis at this time point (Additional file 6) had -log(p) < 4 and, thus, had low statistical significance. Receptor tyrosine kinases and cytosolic kinases showing increased phosphorylation activity in the identified pathways included Epo-receptor, lymphocyte-specific protein tyrosine kinase (Lck), cell division protein kinase 2 (Cdk2), Ser/Thr kinase Nek2 and vascular endothelial growth factor receptor 1 (VEGFR1). The phosphorylation of target peptides by Lck, Nek2 and VEGFR1 was clearly increased, whereas Cdk2 and Epo-receptor showed lesser extents of activation.

It is noteworthy to mention that for five kinases, the dexmedetomidine-induced activation of α_2B_-adrenoceptors first resulted in decreased phosphorylation at the 30 min time point with increased phosphorylation detected at 24 h. These kinases included Fyn-related kinase (Frk), tyrosine kinase Tec, platelet endothelial cell adhesion molecule (PECAM), Cdk2 and Epo-receptor (Fig. 6a).

#### Putative upstream kinase analysis

The canonical pathway analysis performed in GeneGo disregards different phosphorylation sites in the same protein. Therefore, an additional upstream kinase analysis was performed using a computational tool developed by PamGene that is able to link differentially phosphorylated peptides to the upstream kinases that might be responsible for their phosphorylation. Putative PTK and STK upstream kinases are presented in Additional file 7.

At 30 min, an overall decrease in kinase signaling due to dexmedetomidine-evoked α_2B_-adrenoceptor activation was detected. Most specific putative upstream tyrosine kinases included c-Src kinase (CSK), Src family kinase Fgr, hepatocyte growth factor receptor (HGFR; c-MET), EGFR, tyrosine kinase ABL2 (also known as Arg) and receptor tyrosine kinase ErbB-2. In addition to Fgr, all other eight members of the Src family kinases were among the putative upstream kinases (Lyn, Blk, Hck, Src, Fyn, Yes, Frk and Lck). Also many receptor tyrosine kinases were included: ErbB-3 and ErbB-4, fibroblast growth factor receptors 1-4 (FGFR1-4), vascular endothelial growth factor receptors 2 and 3 (VEGFR-2 = KDR; VEGFR-3 = Flt4) and tropomyosin receptor kinases A, B and C. In addition, tyrosine kinase ABL1 and all Tec family kinases (Tec, Btk, Itk, Bmx, Txk) appeared in the list of putative upstream tyrosine kinases. The most specific putative upstream serine/threonine kinases included atrial natriuretic peptide receptors A and B (ANPa, ANPb), serum/glucocorticoid regulated kinases (Sgk, Sgk2, Sgk3), G protein-coupled receptor kinase 1 (Grk1, RHOK), inhibitor of nuclear factor kappa-B kinases beta and epsilon and extracellular-signal-regulated kinase 5 (ERK5). Nine isozymes of the PKC family (α, β1, γ, δ, ι, η, θ, ξ) and five cyclin-dependent kinases (Cdk1, Cdk2, Cdk3, Cdk6, Cdk7) were among the putative upstream Ser/Thr kinases. In addition, many members of the MAPK signaling pathways, including Raf-1, MEK1/2, ERK1/2, p38 kinases and c-Jun N-terminal kinases (JNKs), appeared on the list of putative upstream Ser/Thr kinases.

After 24 h of dexmedetomidine stimulation, slight activation of kinase signaling was observed. At this later time point, putative upstream tyrosine kinases included many kinases that showed decreased activity at the earlier time points: ABL1 and ABL2, Tec family kinases Bmx and Ltk, receptor tyrosine kinases HGFR, EGFR and VEGFR-2 and several Src kinase family members (Frk, Hck, Yes, Src). Similar changes in kinase activity from decreased activity at earlier time points to increased activity at later time points was also observed for certain putative upstream Ser/Thr kinases, such as several PKC isozymes (α, β1, γ, δ, ι, η, θ, ξ), p38 kinases and JNK kinases. In addition to several PKC isoforms, the most specific putative upstream serine/threonine kinases included calcium/calmodulin-dependent protein kinase type IV (CAMK4), serine/threonine-protein kinase H1 (PSKH1), mitogen- and stress-activated protein kinase-1 (MSK1), cyclin-dependent kinase family member CRK7, cyclin-dependent kinase-like 1, 2 and 4 (Cdkl1, Cdkl2, Cdkl4) and the beta adrenergic receptor kinases 1 and 2 (BARK1/Grk2, BARK2/Grk3).

## Discussion

Proliferation and migration of VSMCs are involved in the development of many major cardiovascular diseases, and improved understanding of the mechanisms that control these processes might allow the development of novel approaches to treat various vascular diseases, such as atherosclerosis and restenosis [38]. VSMCs regulate blood vessel diameter, blood pressure and blood flow distribution by their capacity to contract and relax in the vessel wall. Unlike many other cell types, VSMCs retain considerable plasticity even in adult organisms. Differentiated VSMCs express high levels of smooth muscle-specific contractile proteins, such as smooth muscle α-actin and myosin heavy chain. Upon inflammatory stimulation or injury, the cells may de-differentiate to a proliferative state - a phenomenon called phenotypic switching. The proliferative phenotype is characterized by downregulation of smooth muscle differentiation markers and increased production of e.g. extracellular matrix components, cytokines and chemokines [21, 22, 39, 40]. Indeed, VSMCs constantly integrate complex signals present in their local environment, mediated by mechanical forces, neuronal effects, extracellular matrix signals, cytokines and growth factors, and the combination of these signals determines the patterns of gene expression and cell phenotype [40].

α_2_-Adrenoceptor activation may both increase and inhibit cell proliferation, depending on the cell type, and we have previously reported that activation of α_2B_-adrenoceptors greatly enhances the proliferation of A7r5 VSMCs [17]. However, the underlying molecular mechanisms have remained unknown. The aim of this study was to shed light on the mechanisms involved in the α_2B_-adrenoceptor-evoked enhancement of proliferation in A7r5 VSMCs by exploiting DNA and kinase activity profiling microarrays and kinase/phosphatase inhibitor library screening. By combining such different types of screening assays, we aimed to sketch a general overview of the intracellular mechanisms involved in the generation of the α_2B_-adrenoceptor-evoked proliferation response of A7r5 VSMCs.

### DNA microarray: Differentially expressed genes in the regulation of VSMC proliferation

We employed whole-genome microarray analysis and the selective α_2_-adrenoceptor agonist dexmedetomidine to identify differentially expressed genes that are involved in the α_2B_-adrenoceptor-evoked proliferation response of A7r5-α_2B_ cells. Altogether 55 genes were differentially expressed upon α_2B_-adrenoceptor activation; approximately half of these were upregulated and half were downregulated. Although signaling and regulation of α_2_-adrenoceptors has been extensively investigated in other cell types [8, 41–51] and to some extent also in VSMCs [17, 52–58], less is known about how α_2_-adrenoceptor activation modulates gene expression in different types of cells, let alone VSMCs. Four differentially expressed genes from the microarray experiment were selected to be verified by means of quantitative RT-PCR. We were able to verify the altered expression levels of Glrx and Cx3cl1, representing the extremes of the FC spectrum in the microarray data, but genes with small microarray FC values did not show differential expression in the qRT-PCR experiments. It is well known that for microarray expression changes <1.4-fold, microarray and qPCR results do not always agree [59].

Only few reports exist on the effects of adrenoceptor activation on gene expression in VSMCs. Activation of α_1_- and β-adrenoceptors altered gene expression in A7r5 cells; 85% and 75% of the regulated genes displayed decreased expression, respectively. Both α_1_- and β-adrenoceptors inhibited the proliferation of A7r5 cells, but the microarray results indicated different mechanisms for inhibition of cell proliferation: α_1_-adrenoceptor activation induced expression of metabolic genes, whereas β-adrenoceptor activation changed the expression of genes encoding signaling and structural proteins [60]. In contrast, activation of α_1_-adrenoceptors increased the proliferation of primary rat aortic VSMCs [61]. These opposing effects may have been caused by different α_1_-adrenoceptor subtypes present in these systems, or different receptor signaling in the two VSMC models. In our study, activation of α_2B_-adrenoceptors led to increased DNA synthesis and proliferation of A7r5 cells, in association with up- or downregulation of distinct sets of genes. Thus, different members of the adrenoceptor family appear to regulate multiple aspects of biological functions in VSMCs, and their activation can induce opposing effects.

The up- and downregulated gene lists derived from our microarray data were further analyzed using the functional annotation tools GeneFuncster and Ingenuity® pathway analysis. The aim was to identify enriched biological processes, significant networks and upstream regulators related to the differentially expressed genes. The GeneFuncster (GF) analysis yielded more than 200 significantly enriched biological processes; 25% of these were related to cardiovascular system development, cell proliferation, migration, adhesion, cell cycle and vasoconstriction. Also the IPA core analysis detected similar functions specifically related to SMCs, including migration, adhesion, proliferation and contraction.

Both functional analyses identified essentially the same genes to be involved in these functions. Next, we contemplate the roles of individual genes in VSMC proliferation and related functions. For a majority of the regulated genes, the direction of change in expression and the observed effect on cell proliferation were in line with what has been published in the literature. These genes included Egr1, F3, Ptgs2, cdc20, Serpine1, Kif20a, ube2c, Diaph3, Cav1, Rhoa, Nppb and Tpm1. For some genes, however, the observed change in expression and the associated effect on proliferation differed from previously reported effects. These genes included Glrx, Cx3cl1, ccl2, ICAM-1 and Prrx1.

Interestingly, the most highly upregulated gene in our dataset, Ly6/PLAUR domain containing 8 (Lypd8; FC = 2.01, *p* = 4.2 × 10^−6^) did not receive any annotations in the functional analyses. Lypd8 is a member of the Ly6/PLAUR family of glycophosphatidylinositol-anchored cell surface proteins with immunity-related roles [62]. Other members of the Ly6/PLAUR family have been shown to be involved with proliferation of neutrophils [63] and to be able to activate transcription factors like activator protein 1 (AP-1) [64], which regulates gene expression and controls a number of cellular processes including differentiation, proliferation, and apoptosis [65]. However, no studies on the possible functions of Lypd8 existed, until very recently, when Okumura et al. showed that Lypd8 is selectively expressed in intestinal epithelial cells and its product is capable of preventing flagellated bacteria from invading the colonic epithelium in mice [66]. Our microarray results clearly indicated that Lypd8 expression was upregulated in A7r5 VSMCs upon activation of α_2B_-adrenoceptors, suggesting that the functions of this gene would not be restricted solely to the intestine. However, the possible role of Lypd8 in the regulation of A7r5 VSMC proliferation remains to be elucidated.

According to our results, upregulation of Egr1, F3, Ptgs2, cdc20, Serpine1, Kif20a, ube2c and Diaph3 was associated with α_2B_-adrenoceptor-evoked proliferation of A7r5-α_2B_ cells. All of these genes have previously been implicated in the regulation of VSMC proliferation and also proliferation of several non-vascular cell types. In line with our results, Egr1 seems to be essential for proliferation in many, but not all, cell types [67] and its expression levels often closely correlate with cell proliferation [68–70]. Increased Egr1 expression is detected in different vascular pathophysiological processes which involve increased vascular cell proliferation [20] and Egr1 seems to be crucial for effective vascular cell cycle progression in arteriogenesis [71]. Tissue factor F3 is constitutively expressed in VSMCs but it can be upregulated by growth factors and other stimuli [72]. It may contribute to cardiovascular diseases e.g. by inducing proliferation and migration of VSMCs [73–75]. VSMC proliferation has been associated with upregulated F3 expression [76], whereas F3 knockdown has led to inhibition of proliferation and increased apoptosis [77]. Ptgs2 directly promotes VSMC proliferation through upregulated gene expression [78], but also by mediating growth-promoting responses to such compounds as angiotensin II and tumor necrosis factor [79–81]. Consistently, decreased expression of Ptgs2 is associated with decreased VSMC proliferation and inhibition of cell migration [82–84]. Cdc20 is an important cell cycle regulator for the completion of mitosis. In many cancer cell lines, its knockdown inhibits cell division/proliferation, and cdc20 overexpression greatly promotes cell division/proliferation [85, 86]. Also in proliferating human mesenchymal stem cells, cdc20 was upregulated by more than three-fold [87]. So far, little is known about the effects of cdc20 expression on the proliferation of VSMCs. Our results now suggest that increased cdc20 expression could have a growth-promoting effect also in VSMCs. In the literature, a rather uniform impression exists that Serpine1 expression promotes the proliferation of VSMCs [88–93], which is in good agreement with our results.

Also Kif20a, ube2c and Diaph3 were upregulated in association with increased A7r5 cell proliferation, but these genes did not receive many annotations in the functional analyses. Interestingly, in proliferating human mesenchymal stem cells, selected cell cycle-related genes that were upregulated more than three-fold included Kif20a, ube2c and cdc20 [87] and, in sheep carotid arteries, ube2c and Diaph3 were highly upregulated during early life and were associated with growth and proliferation [94]. Knockdown of Kif20a suppressed the proliferation of different cancer cell lines [95, 96]. Silencing of ube2c inhibited VSMC proliferation, whereas increased ube2c levels increased VSMC proliferation [97].

The proliferation of VSMCs is not only enhanced by upregulation of growth-promoting genes but also by downregulation of genes with antiproliferative effects. Nppb, Cav1, Tpm1 and Rhoa represent such genes in our dataset; the decreased expression of these genes was associated with increased proliferation of A7r5-α_2B_ cells. Natriuretic peptides, including Nppb, have potent antiproliferative and antimigratory effects on VSMCs [98–101], which supports our observation of downregulated Nppb in response to α_2B_-adrenoceptor activation and the concomitant increase in A7r5-α_2B_ cell proliferation. Also, decreased Cav1 expression has been reported to be associated with proliferating VSMCs [102–106] and Tpm1 has been shown to be involved in the microRNA-induced proliferation of VSMCs; increased expression of Tpm1 has inhibited VSMC proliferation [107]. In line with our observations of decreased Rhoa expression and increased cell proliferation is the observation of Tseliou et al. who showed that knockdown of Rhoa in cytomegalovirus-infected human cell lines restored their proliferation rate [108]. However, in contrast with our results, there are many studies reporting that suppression or downregulation of Rhoa leads to inhibited proliferation of VSMCs [109–114]. These differences may perhaps be attributed to the existence of many different environmental signals affecting Rhoa signaling and to inherent differences in Rhoa signaling in different cell types [115]. Moreover, a common feature of the Cav1, Tpm1 and Rhoa genes is that their expression has been related to the differentiated, contractile phenotype of VSMCs rather than the proliferative, noncontractile VSMC phenotype [106, 107, 115–120]. Caveolae are less abundant in proliferating SMCs than in contractile, nonproliferating cells [121], and Cav1 is more likely to mediate contractile as opposed to proliferative stimulation in smooth muscle [117, 122–125]. Rhoa and Tpm1 seem to be important in regulating VSMC contraction and actin reorganization [38, 119, 126, 127], which are functions of the differentiated, nonproliferative phenotype. Tpm1 has even been suggested to be a better phenotypic marker for quiescent VSMCs than the traditional markers smooth muscle α-actin and myosin heavy chain [107, 120]. Considering all this, it is reasonable that these genes are downregulated in proliferating VSMCs as seen in our study.

Thus, for most of the differentially regulated genes associated with α_2B_-adrenoceptor-evoked proliferation of A7r5 VSMCs, there is strong evidence in the published literature supporting our results. However, for Glrx, Cx3cl1, ccl2, ICAM-1 and Prrx1, our observations differ from what has been previously reported. For Glrx and Cx3cl1, up- and downregulation, respectively, were even confirmed with RT-PCR. Contrary to our results, where upregulation of Glrx was associated with increased proliferation of A7r5 VSMCs, upregulation of Glrx has been linked to decreased proliferation of A7r5 VSMCs, as reported by Urata et al. [128]. In pulmonary artery SMCs and in human lung cancer tissue, Glrx expression showed an inverse correlation with proliferation [129, 130]. At the same time, Glrx may play a role in protecting cells from apoptosis [131]; therefore, its role in cardiovascular functions may not be entirely straightforward, as responses may be dependent upon cell type and extracellular stimuli [132]. This may explain the differences between our results and the literature.

In our results, downregulation of Cx3cl1, ccl2, ICAM-1 and Prrx1 was associated with increased proliferation. Contrary to our results, there is solid evidence indicating that Cx3cl1 and ccl2 may induce VSMC proliferation [133–144]. Until now, there is only one report suggesting that ccl2 inhibits VSMC proliferation [145]. Increased expression of ICAM-1 has been associated with increased proliferation of microvascular endothelial cells [146], and it may also promote the proliferation of fetal VSMCs [147]. On the other hand, it has also been reported that ICAM-1 does not contribute to SMC proliferation [148]. Similarly, overexpression of Prrx1 has been reported to promote VSMC proliferation [149, 150]. Cx3cl1, ccl2 and ICAM-1 are induced by inflammatory cytokines [133, 146, 151–155]. The pro-proliferative effects of these genes/proteins may be mediated through a pro-inflammatory signaling pathway, whereas the α_2B_-adrenoceptor-evoked proliferation response, as seen in this study, would be mediated through another mechanism not requiring Cx3cl1 or ccl2. Furthermore, increased expression of ICAM-1 has been detected in differentiated contractile VSMCs [156], and Prrx1 has been shown to regulate SMC differentiation marker genes in fully differentiated VSMCs [115, 157–159]. This is in line with these genes being downregulated in proliferating VSMCs, as seen in our study.

Some of the observed effects may have been indirect and evoked by interactions of the differentially expressed genes altering the expression of other genes. For example, Egr1 is an important activator of various proliferation-associated genes such as cdc20 [160, 161], and consistent with this, both Egr1 and cdc20 were upregulated in our results. Furthermore, increased expression of Glrx or Ptgs2 can limit the expression of ICAM-1 in VSMCs [131, 153], and in line with this, Glrx and Ptgs2 were upregulated and ICAM-1 was downregulated in our experiment.

Classically, α_2B_-adrenoceptors couple to inhibitory G_i/o_-type G-proteins leading to inhibition of adenylyl cyclase activity and decreased cyclic AMP (cAMP) levels [162], but this receptor is also capable of coupling to stimulatory G_s_-proteins, activating adenylyl cyclases and increasing cAMP levels [50, 163]. Some of the differentially regulated genes may be regulated by cAMP. cAMP can inhibit VSMC proliferation by inhibiting Egr1 expression [20] or the expression of other pro-mitogenic genes by inhibiting Rhoa activity [164]. Ptgs2 induction is known to regulate VSMC proliferation but the effect depends on several factors including the coupling of cAMP to either growth-inhibitory or growth-promoting pathways [165]. Decreased cAMP levels could provide an explanation for the upregulation of Egr1 and Ptgs2 and the concomitant increase in A7r5 VSMC proliferation, but this explanation would not be applicable for Rhoa.

### Kinase/phosphatase inhibitor library screening

Protein phosphorylation regulates most processes in eukaryotic cells, and abnormal phosphorylation often is a cause or consequence of disease. Reversible protein phosphorylation requires not only protein kinases to phosphorylate specific serine, threonine or tyrosine residues of target proteins but also protein phosphatases to remove these phosphates [166]. Protein kinases form a big group of structurally related enzymes that participate in mediation of signal transduction in virtually all cellular processes, including cell growth and differentiation [167]. To explore the signaling pathways mediating α_2B_-adrenoceptor-evoked VSMC proliferation, A7r5-α_2B_ cells were subjected to screening with an 84-compound chemical library consisting of commercially available kinase and phosphatase inhibitors. The effects of different inhibitors on α_2B_-adrenoceptor-evoked cell proliferation were monitored with a method using BrdU incorporation as readout [168], an assay that measures the amount of newly synthetized DNA [169].

Issues that complicate the use of commercially available enzyme inhibitors may include poor solubility, general toxicity and lack of specificity [170], and inhibitors may give inconsistent results because of the complexity of the involved pathways. Still, inhibitors are valuable tools to study the biology and therapeutic potential of specific kinases. In addition to the limited selectivity of many kinase inhibitors, intracellular signaling pathways contain such redundancy that single drugs might not be able to overcome the robustness of biological networks [171, 172]. A given receptor may mediate its effects through several different signaling mechanisms, and parallel mechanisms may compensate for the effects of a blocked signaling route. This might provide an explanation also in our case, where no single mechanism could be pinpointed as the key regulator of the drug effects. BrdU incorporation was inhibited by less than 30% even with the most effective inhibitors.

We were able to identify several compounds that had statistically significant inhibitory effects on the dexmedetomidine-evoked proliferation of A7r5-α_2B_ cells. We identified several kinases and phosphatases that have been implicated in the promotion of VSMC proliferation, which gives biological credibility to our results. Kinases and phosphatases possibly promoting the mechanisms by which α_2B_-adrenoceptor activation leads to increased proliferation of A7r5 VSMCs included calcineurin [61, 173–175], protein kinase CK2 (casein kinase) [176], Src kinases [177–182], JNK [183, 184], p38 MAP kinase [185–187], Cdk1/2 [85, 188–191], PI3-kinases [173, 192–195], EGFR [179, 196–199] and Raf-1 kinase [37, 177, 200]. A more detailed description of the effects of these kinases on VSMC proliferation is summarized in Additional file 5. Our results underline the importance of kinases and their interactions in the regulation of VSMC proliferation, since it was impossible for any single inhibitor to overcome the robustness of the cellular signaling networks and completely block the proliferation response.

### Kinase activity profiling

Information about substrate proteins is required to integrate kinases into their biological signaling networks [201, 202]. Peptide arrays that monitor kinase activity in cell lysates can be used for the analysis of the kinome and for drug screening purposes [202, 203]. Spotting consensus substrate peptides for kinases on a solid support, incubation with cell lysates and detection with radioactive or fluorescent antibodies makes it possible to determine the kinases that are active in the assay system. Multiplexed treatment of peptide microarrays with cell lysates generates snapshots of the actual phosphorylation equilibrium within cells and reflects the activity of kinases and phosphatases. This should facilitate novel approaches based on phosphorylation fingerprints [202].

To screen for kinases participating in the α_2B_-adrenoceptor-mediated regulation of VSMC proliferation, we performed kinase activity profiling experiments with PamChip microarrays for protein tyrosine kinases (PTK) and serine/threonine kinases (STK), consisting of altogether 288 target peptide sequences (Additional file 8). We aimed to explore both primary kinase responses at time points < 30 min and secondary kinase responses at later time points after stimulation of A7r5-α_2B_ cells with dexmedetomidine. Treatment effects on kinase signaling were most pronounced at 30 min, where decreased phosphorylation of altogether 40 peptides (36 and 4 on the PTK and STK chips, respectively) was seen. We postulate that at the 30 min time point the A7r5-α_2B_ cells may be in a state of metabolic perturbation, still recovering from contraction caused by dexmedetomidine-induced activation of α_2B_-adrenoceptors. α_2B_-adrenoceptor activation leads to rapid and transient myosin light chain (MLC) phosphorylation at Ser19 (pMLC), which is the hallmark biochemical event leading to contraction, peaking at 20-45 s and returning to baseline levels by 2 minutes [58]. However, when the contraction-inducing agonist is not removed, pMLC can be further phosphorylated at Thr18 (ppMLC) [204–209]. MLC diphosphorylation does not increase the contractile force but it slows down the relaxation of arterial smooth muscle [210]. By means of live cell microscopy, we have observed that A7r5 VSMC contractions may last at least 20 min when the contraction-inducing agonist is not removed. Additional files 9 and 10 contain time-lapse movies showing typical time-courses of vasopressin-induced contraction of wild-type A7r5 cells and their relaxation after removal of the agonist. This could provide a possible explanation for the overall decrease in kinase activity at 30 min. Thus, the observed overall inhibition of kinase activity might not be directly evoked by α_2B_-adrenoceptor activation, but is more likely to represent indirect effects caused by other signaling mechanisms.

By 2 h, no differences in kinase activities between dexmedetomidine-stimulated and vehicle-treated cells could be observed, suggesting that by this time the cells had recovered from the contraction and returned to a basal state. Still later, after 24 h of exposure to dexmedetomidine, kinase activities were increased to some extent compared to baseline. However, it remains unclear whether the peak of kinase activity had taken place between 2 h and 24 h, or whether we saw the beginning of increasing overall kinase activity at 24 h. The stability of dexmedetomidine in cell culture medium over a period of 24 h was confirmed by means of quantitative mass spectrometry. In retrospect, the selected time points might not have been optimal. Additional time points at e.g. 12 h and 36 h could have provided an answer to this question.

Only six out of 144 peptides on the STK chip showed differential phosphorylation after treatment with dexmedetomidine. This paucity of effects on Ser/Thr versus tyrosine kinase activities was not unexpected; tyrosine kinase signaling, as monitored by the PTK chip, is more typical for neurotransmitter receptors than Ser/Thr signaling [182, 211, 212]. Although GPCRs do not use tyrosine phosphorylation as a direct mechanism of transmembrane signaling, activation of downstream non-receptor tyrosine kinases upon GPCR activation is important for mitogenic signaling [211, 212]. The subsequent canonical pathway and upstream kinase analyses were thus mostly driven by the kinase activity results obtained from the PTK chip, but also the STK chip results were included. Canonical pathway analysis revealed ten statistically highly significant pathways (−log(p) < 7) at the 30 min time point, but at the 24 h time point, no pathway reached a -log(p) value <4, and thus, their significance is questionable. Therefore, we focus on discussing the significant pathways detected at *t* = 30 min. Canonical pathway maps are based on recognized connections between signaling pathway components and other pathways appearing in particular cell types and represent a set of signaling and metabolic maps in a comprehensive manner [213]. The traditional views of signaling pathways have been challenged by at least two recent developments; first, a massive increase in the number of components linked to a particular pathway, and second, an appreciation of the variable quantitative contributions of each of these components to dynamic signal propagation [213]. Signal transduction analysis should perhaps pay more attention to recurring signaling cascades in different pathway maps rather than to try to identify entire pathways where only a few kinases seem to be involved.

The c-Raf-1-MEK1-MEK2 signaling cascade appeared in most of the top ten significant pathways; c-Raf-1 phosphorylates MEK1 and MEK2, and dexmedetomidine treatment (30 min) decreased the activity of these kinases. The c-Raf-1-MEK-ERK pathway is one of the best characterized MAP kinase signaling pathways known to regulate cell proliferation [37]. Inhibition of this pathway after 30 min of dexmedetomidine exposure gives support to our hypothesis of the cells being in a state of metabolic perturbation after the initial α_2B_-adrenoceptor-evoked contraction. Interactions of PLCγ with the PDGF receptor or Epo-receptor, LAT or Syk appeared in many of the significant pathways. In addition, EGFR and the kinases PDK and Fer appeared in many of the significant pathways. It is known that many receptor tyrosine kinases, including PDGFR, Epo-receptor and EGFR, directly phosphorylate and activate PLCγ [214–217]. Also, interactions with LAT [218] and Syk [219, 220] have been shown to be required for PLCγ activation. In our peptide array, 30 min exposure to dexmedetomidine significantly decreased the phosphorylation activity of all of these receptor tyrosine kinases and cytosolic protein tyrosine kinases. Actually, it has been shown that α_2_-adrenoceptors can activate PLC signaling, but such PLC activation is not in itself sufficient to induce a mitogenic response. Preceding activation of Na^+^/H^+^ exchange and early gene expression may be involved [14, 221]. Although the PTK chip did not contain a peptide substrate for Lyn kinase, it appears that Lyn kinase activity was also modulated by dexmedetomidine, because Syk, LAT and PLCγ are successive downstream targets in the Lyn signaling cascade (Immune response_Fc epsilon RI pathway, see Additional file 11) and all of these downstream targets showed decreased activity after dexmedetomidine treatment. Moreover, Lyn was also identified as a putative upstream kinase in the upstream kinase analysis, which utilizes different databases than the canonical pathway analysis, includes known phosphorylation sites and is altogether more specific. The finding that different database approaches point in the same direction gives more confidence in the results, and inhibition of Lyn kinase activity appears to affect many downstream kinases.

Five tyrosine kinases, i.e. Frk, Tec, PECAM-1, Cdk2 and Epo-receptor, that were first inhibited after 30 min dexmedetomidine treatment showed increased activity after 24 h of dexmedetomidine stimulation. All five have been implicated in promoting proliferation of different types of cells [191, 222–228]. In our system, these kinases showed distinct temporal regulation patterns: inhibition during a suggested initial phase of metabolic perturbation (*t* ≤ 30 min), followed by increased activity when the cells had reached a phase of active proliferation (*t* = 24 h).

To further explore the possible signaling mechanisms involved in α_2B_-adrenoceptor-evoked proliferation of A7r5-α_2B_ VSMCs, a putative upstream kinase analysis was performed to link differentially phosphorylated peptides to the upstream kinases possibly causing the phosphorylation. Several of the upstream kinases that showed decreased activity at 30 min were identified as putative upstream kinases also at 24 h when activation of kinase signaling was detected. These kinases included the receptor kinases HGFR (MET), EGFR, VEGFR-1 (Flt1) and VEGFR-2 (KDR), and the cytosolic kinases ABL2 and Bmx, which also had the highest specificity scores (Additional file 7) among the putative upstream tyrosine kinases. ABL2 promotes proliferation of breast cancer cells and recombinant 293 T cells [229, 230], and Bmx is involved in angiogenesis [231] and in regulation of the actin cytoskeleton and cell motility [232]. Based on our results, these kinases may have similar roles in the regulation of VSMC proliferation. Putative upstream Ser/Thr kinases with the highest specificity scores included CaMK4 and serine/threonine protein kinase 1 (PSKH1), both of which have been implicated in the regulation of cell proliferation [233–235]. Moreover, CaMK4 has been identified as an important regulator of pulmonary artery vascular remodeling [236]. Thus, our finding of CaMK4 involvement in regulation of VSMC proliferation derives support from previous observations.

We identified several kinases that have been implicated in regulation of cell growth and proliferation, which gives biological significance to our study. The following growth factors and growth factor receptors appeared as putative upstream regulators in the DNA microarray and/or kinase activity profiling data analyses: bFGF, PDGF, EGFR, HGFR, VEGFR-1 and VEGFR-2 (KDR). PDGF, bFGF and HGF were identified as potential upstream regulators of 7, 9 and 11 genes in our dataset, respectively. EGFR, HGFR, as well as VEGFR-1 and VEGFR-2, were among the top ten putative upstream kinases (showing increased activity at 24 h) in the upstream kinase analysis. Moreover, EGFR was also identified as a potential target kinase in the inhibitor library screening. It not unexpected that growth factors and growth factor receptors appeared among the upstream regulators in the signaling pathways related to promotion of proliferation.

While bFGF and PDGF are known to induce VSMC proliferation [237–240], little is known about the possible interactions of α_2B_-adrenoceptors with bFGF- or PDGF-dependent mechanisms. β-adrenoceptor activation has increased the expression of both of these growth factors [241]. AngII promotes VSMC hypertrophy and endogenous bFGF expression, but increased bFGF expression alone was not sufficient to stimulate VSMC growth; also PKC activation was required [242]. EGFR transactivation by GPCRs can activate multiple mitogenic pathways. Mediators such as Src kinases, Ca^2+^, PKC and PKA may participate in this process, and especially activation of the EGFR-MAPK pathway is commonly involved in regulation of gene expression and cell proliferation [243, 244]. α_2_-Adrenoceptors are capable of activating MAP kinases through EGFR transactivation in different cell types including astrocytes [245], renal tubular cells [12], intestinal epithelial cells [45], Müller cells [246] and PC12 cells [46]. The intensity and duration of EGFR transactivation and subsequent MAP kinase activation may control the switching between cell proliferation and alternative fates such as differentiation [247–250]. HGF and its receptor HGFR regulate normal cell growth and development in many tissue types, but they also control growth, invasion and metastasis of cancer cells [251–254]. HGFR may be transactivated by both EGFR and many GPCRs [255]. HGF/HGFR signaling is activated in angiogenesis [254], and may be involved in the pathogenesis of atherosclerosis and restenosis [256]; HGFR is expressed on VSMCs isolated from atherosclerotic plaques and it triggers signaling cascades (involving PI3-kinases, Akt, MEK and Erk1/2) mediating migration of VSMCs [256, 257]. The VEGF/VEGFR signaling pathway, and especially VEGFR-2, is vital for the induction of angiogenesis and drives both endothelial cell proliferation and migration [258–260]. VEGF/VEGFR signaling may have complex effects on VSMC proliferation: a VEGF-mediated pathway has been implicated in the promotion of VSMC proliferation [261, 262], but VEGF/VEGFR-2 has also been identified as a negative regulator of VSMCs [260]. VEGF also appears to be a potent stimulator of VSMC migration [263]. Both α_2_- and β-adrenoceptors have been reported to regulate VEGF/VEGFR expression [264]. VEGFR-1 is coexpressed with α_2B_-adrenoceptors in the mouse placenta and, in a knockout mouse model, deletion of the gene encoding α_2B_-adrenoceptors resulted in upregulation of VEGFR-1 and severely impaired placental angiogenesis [265]. VEGFR-1 has been identified as an antiangiogenic regulator, binding VEGF with high affinity and preventing it from activating other receptor subtypes, including VEGFR-2 [266]. Based on the results of our upstream regulator analyses, many growth factors and growth factor receptors appear to be activated upon sustained (24 h) exposure to dexmedetomidine. The results suggest that α_2B_-adrenoceptors may mediate their pro-proliferative effects in A7r5 VSMCs by promoting the activity of endogenous bFGF and PDGF and the growth factor receptors EGFR, HGFR and VEGFR-1/2.

Even if our analysis found weaker evidence for Ser/Thr kinase involvement in the regulation of VSMC proliferation by α_2B_-adrenoceptors than for tyrosine kinase involvement, the putative identified upstream Ser/Thr kinases included relevant actors such as the PKC isoforms δ, γ and Ɛ and the beta adrenergic receptor kinases 1 and 2 (BARK1/Grk2, BARK2/Grk3). These, too, may be involved in the observed activation of kinase signaling after 24 h of exposure to dexmedetomidine. PKC activity modulates several processes in VSMCs, including contraction, growth and proliferation [267–269]. PKC has been shown to exert both proliferative and antiproliferative effects on cultured VSMCs [267]. Activation of PKC and, concomitantly, Erk1/2 has been implicated in VSMC proliferation induced by different stimuli, such as AngII [270–272], high glucose [273], PDGF-BB and noradrenaline [274]. Several PKC isoforms have been identified in VSMCs. The opposing effects of different PKC isoforms [267, 268, 275–279] emphasize the complexity of regulation of VSMC proliferation. A possible mechanism related to the proliferation response caused by α_2B_-adrenoceptor activation could be mediated through pathways involving regulating the activity of PKC isoforms. Moreover, IPA identified PKC isoforms as potential upstream regulators of gene expression upon α_2B_-adrenoceptor activation (Fig. 4B). Six genes in our gene array dataset were identified as possibly regulated by PKC isoforms, and for five of these, the observed change in gene expression was in the same direction as predicted.

GPCR signaling may be attenuated by Grk2-mediated desensitization. Also, Grk2 appears to inhibit VSMC proliferation responses to many GPCR agonists and also to PDGF, but not EGFR-mediated VSMC proliferation [280, 281]. Prolonged exposure of α_2B_-adrenoceptors to agonists results in downregulation of the receptors [282], mediated by both Grk2 and Grk3 [47]. Although little is known of the effects of Grk3 on VSMC proliferation, it has been established that Grk3 plays an important role in the survival and proliferation of metastatic prostate cancer cells and in stimulation of tumor angiogenesis [283, 284]. Based on this, we are tempted to speculate that the signaling mechanisms of the α_2B_-adrenoceptor-evoked proliferation response in A7r5 VSMCs could involve transactivation of EGFR, which is not affected by the inhibitory effects of Grk2, and that increased activities of Grk2 and Grk3 after 24 h of dexmedetomidine treatment could be a counteracting mechanism leading to α_2B_-adrenoceptor downregulation and attenuation of the α_2B_-adrenoceptor-evoked proliferation response.

### Comparison of the employed screening methods

A secondary aim of the present study was to evaluate the suitability of three different in vitro screening approaches to investigate gene expression patterns and intracellular signaling pathways related to α_2B_-adrenoceptor-evoked VSMC proliferation. We identified several gene products/kinases that appeared as top hits in two out of the three screens. HGF/HGFR and isoforms of PKC appeared as hits in the DNA microarray and in the kinase activity profiling. Raf-1, EGFR, Src kinases and the MAP kinases p38 and JNK appeared as hits in the inhibitor library screening and in the kinase activity profiling. The roles of HGF/HGFR, PKC and EGFR in the regulation of VSMC proliferation have already been discussed. It is well established that also Raf-1, Src kinases, p38 and JNK kinases participate in the regulation of VSMC proliferation [177, 178, 183, 185–187, 285, 286], but due to space limitations we will not discuss this in more detail here. That these kinases were identified as hits by two different screening methods increases the confidence level of the results. However, these results are only preliminary and should be regarded as starting points for more detailed analysis employing cell models, kinase inhibitors and different cell-based assays.

Each of the employed screening methods has its own advantages and disadvantages. Gene microarrays enable the analysis of mRNA expression levels and are of significant value for the elucidation of molecular mechanisms that govern cellular physiology [201]. Still, very critical transcripts may be expressed at low levels [287, 288] and small changes in gene expression can lead to large changes in the cell phenotype and function. Indeed, a comprehensive description of cellular metabolism and intracellular signaling might provide more useful information than gene expression analysis alone. To some extent, monitoring of cellular functions can be achieved by kinase activity analysis. Peptide microarrays have emerged as promising tools for the analysis of protein functions and kinase activity [31, 33, 203, 289–291]. The employed PamChip kinase activity profiling technology is relatively new. The kinase substrates on the PamChips are peptides consisting of 13–14 amino acids and may have similarities with other substrates, which makes cross-reactivity possible. However, other investigators have validated their PamChip results by Western blot analysis and demonstrated that kinase inhibitors affected peptide array phosphorylation patterns consistently with the expected actions of these inhibitors [292, 293].

In the present study, DNA microarray and kinase activity profiling were technically successful and of high experimental quality. As for the kinase/phosphatase inhibitor library screening, the obtained results showed large variability and, consequently, the screen failed to reach a z’ score of 0.5, indicating limited robustness. Nevertheless, also the inhibitor library screen has advantages; many different readouts can be employed to evaluate inhibitor effects and data analysis is very straightforward. We employed α_2B_-adrenoceptor-evoked cell proliferation as the readout for evaluating kinase inhibitor effects by measuring BrdU incorporation. Individual test compounds did not have very large inhibitory effects on the proliferation response, demonstrating that compensatory signaling mechanisms exist and that it may be difficult for a single kinase inhibitor to overcome the robustness of biological networks. Furthermore, kinase inhibitors often lack specificity and a kinase inhibitor will often inhibit multiple related proteins making it difficult or impossible to define the specific functions of an individual target [294]. Hence, the goal of inhibitor screening in general should not be full reconstruction of a regulatory network, but rather to identify a set of kinases linked to a specific response in a given cell line. Then, the hypotheses derived from results such as those presented here need to be validated by further experiments with different inhibitors for the same target or with alternative methods, e.g. using siRNA silencing technology [172].

Peptide arrays, such as the PamChip kinase activity profiling technology, represent a more comprehensive approach to the investigation of kinase signaling compared to traditional genetic and biochemical approaches, which for technical and practical reasons are typically pursued for one gene or pathway at a time. Moreover, peptide arrays of kinase-specific substrates are incubated with cell lysates, allowing analysis of cellular signaling without a priori assumptions of the pathways possibly influenced [295]. Their simplicity and high throughput make peptide microarrays especially suitable for practical applications; monitoring of drug effects on cellular kinomics as a tool for drug development, and for kinomics-based diagnostics and prognosis evaluation of diseases [296]. That eukaryotic protein kinases form a large superfamily of homologous proteins and that their kinase domains (catalytic domains) are fairly conserved [297] makes peptide arrays suitable for the analysis of cell lysates independent of their origin and species. This is a great advantage compared to DNA microarrays, where arrays are performed on species-specific chips.

## Conclusions

α_2B_-adrenoceptor activation stimulates VSMC proliferation, and this may be important for the development and plasticity of blood vessels and for the pathology and therapeutics related to different cardiovascular disorders and even neoangiogenesis of tumors. However, the underlying cellular mechanisms and signal transduction pathways have remained mostly unknown. In this study, we employed three different approaches to investigate changes in gene expression, signaling pathways and kinase activation profiles related to α_2B_-adrenoceptor-regulated VSMC proliferation. Based on the results of this study, the cellular mechanisms participating in this proliferation response appear to be complex and include redundancy. Differentially regulated genes that were identified by two different functional analyses (GeneFuncster and IPA) included Egr1, Ptgs2, Cx3cl1, Cav1 and Nppb. This provides further support for the already claimed involvement of these genes in the regulation of VSMC proliferation. Our analysis also suggested the involvement of other genes, such as F3, Serpine1, Rhoa and Prrx1 that are known to play important roles in the regulation of SMC-specific functions including migration, adhesion and contraction. Nevertheless, also some surprise findings were encountered. Indeed, the most highly upregulated gene in our dataset, Ly6/PLAUR domain containing 8 (Lypd8) did not receive any annotations in the functional analyses. Lypd8 belongs to the Ly6/PLAUR family of proteins. Members of the Ly6/PLAUR family activate transcription factors to regulate gene expression and processes such as differentiation, proliferation, and apoptosis. Lypd8 has been reported to be selectively expressed in intestinal epithelial cells, but our microarray results clearly indicated that Lypd8 expression was upregulated in A7r5 VSMCs upon activation of α_2B_-adrenoceptors, suggesting that the functions of this gene might not be restricted to the intestine.

A given receptor may mediate its effects through several different signaling mechanisms, and parallel mechanisms may compensate for the effects of a blocked signaling route. This redundancy might provide an explanation for our kinase/phosphatase inhibitor results, where no single mechanism could be pinpointed as the key regulator of VSMC proliferation. Agonist-stimulated DNA synthesis was inhibited by less than 30% by the most effective enzyme inhibitors. The results suggested involvement of several kinases that have already been implicated in the promotion of VSMC proliferation. Identified kinases possibly promoting α_2B_-adrenoceptor-mediated VSMC proliferation included protein kinase CK2, Src kinase, JNK, p38 MAP kinase, Cdk1/2, PI3-kinases, EGFR and Raf-1 kinase. The results underline the importance of kinases and their interactions in the regulation of VSMC proliferation. It was impossible to overcome the robustness of the involved biological signaling networks with any single inhibitor and to completely block the proliferation response.

Identified putative upstream tyrosine kinases included the cytosolic kinases ABL2 and Bmx, and several growth factors and growth factor receptors, such as bFGF, PDGF, EGFR, HGFR, VEGFR-1 and VEGFR-2. All of these growth factors/growth factor receptors have been implicated in regulation of VSMC proliferation, which supports the biological significance of the present findings. Our results suggest that α_2B_-adrenoceptors may mediate their pro-proliferative effects in VSMCs by promoting the activity of endogenous bFGF and PDGF and the growth factor receptors EGFR, HGFR and VEGFR-1/2. As a more novel finding, the Src family kinase Lyn was identified by us as a putative upstream kinase. Its involvement was suggested by two different pathway analysis approaches. Lyn is known to be expressed in VSMCs [298, 299], to be regulated by pertussis toxin-insensitive GPCR signaling [300], and has been identified as an important regulator of GPCR trafficking and their effects on cell proliferation [301, 302]. Identified putative Ser/Thr kinases included some biologically relevant kinases such as several PKC isoforms and the β-adrenergic receptor kinases 1 and 2 (Grk2 and Grk3). Cross-talk between the signaling mechanisms involved in α_2B_-adrenoceptor-evoked VSMC proliferation thus appears to involve PKC activation, subsequent changes in gene expression, transactivation of EGFR, and modulation of kinase activities and growth factor-mediated signaling.

Pathway analysis and upstream kinase analysis emerged as useful approaches, as also relatively weak signals could be identified, and they provided a broader overview of the signaling events within the cell compared with targeted experiments on individual genes or kinases. Still, any direct mechanistic conclusions about an observed effect should be made with appropriate caution, as the observed changes could be either causes or consequences of the proliferation response. We conclude that the employed screening tools are useful for hypothesis generation, but hypothesis testing will require additional approaches, such as overexpression cell models or gene silencing with e.g. siRNA technology.

## Additional files

Additional file 1: BioMol kinase and phosphatase inhibitor library; 84 compounds (former CAT# 2831A). (XLSX 610 kb)

Additional file 2: Up- and down-regulated genes in dexmedetomidine-treated vs. vehicle-treated A7r5-α2B cells. (XLSX 34 kb)

Additional file 3: Significantly enriched (*p* < 0.01) cellular component (A) and molecular function (B) GO terms in differentially regulated genes induced by α_2B_-adrenoceptor activation in A7r5-α_2B_ vascular smooth muscle cells determined by GeneFuncster functional enrichment analysis. (DOCX 27 kb)

Additional file 4: Significant biological functions and diseases determined by IPA. (XLSX 45 kb)

Additional file 5: Inhibitors with statistically significant (*p* < 0.01) inhibitory effects on the α2B-adrenoceptor-evoked proliferation in dexmedetomidine-treated vs. vehicle-treated cells. Target kinases/phosphatases of these inhibitors and reported effects of these kinases on cell proliferation are also indicated, if possible. In cases when little data are available inVSMCs, the table will be augmented with information of kinase effects on cell proliferation in other cell types. (XLSX 14 kb)

Additional file 6: Top ten pathways indicated from kinase activity data significantly changed (−log(p) > 4) a *t* = 30 min (A) and at *t* = 24 h (B). (DOCX 195 kb)

Additional file 7: Putative PTK and STK upstream kinases at *t* = 30 min (A = PTK, B = STK) and *t* = 24 h (C = PTK, D = STK). (DOCX 247 kb)

Additional file 8: Full list of target peptides on the STK and PTK chips. (XLSX 38 kb)

Additional file 9: Time-lapse video of wildtype A7r5 vascular smooth muscle cell contraction induced with 100 nM vasopressin perfusion. (PPTX 5531 kb)

Additional file 10: Time-lapse video of washing (Na+/Hepes buffer) wildtype A7r5 vascular smooth muscle cells being after vasopressin stimulation, followed by partial relaxation of the cells. (PPTX 3420 kb)

Additional file 11: Immune response_Fc epsilon RI pathway. Blue thermometers indicate inhibition of kinase activity. (DOCX 666 kb)
