# Supplementary material for: Gene expression profiles and signaling mechanisms in α2B-adrenoceptor-evoked proliferation of vascular smooth muscle cells
Source: BMC Syst Biol. 2017 Jun 28;11:65. doi: 10.1186/s12918-017-0439-8 (PMC5490158; doi:10.1186/s12918-017-0439-8)
Supplement: Supplementary file 3 — Significantly enriched (p < 0.01) cellular component (A) and molecular function (B) GO terms in differentially regulated genes induced by α2B-adrenoceptor activation in A7r5-α2B vascular smooth muscle cells determined by GeneFuncster functional enrichment analysis. (DOCX 27 kb) [file 12918_2017_439_MOESM3_ESM.docx]

**Additional file 3.**

Significantly enriched (p < 0.01) cellular component (A) and molecular function (B) GO terms in differentially regulated genes induced by α_2B_-adrenoceptor activation in A7r5-α_2B_ vascular smooth muscle cells determined by GeneFuncster functional enrichment analysis.
