# Supplementary material for: Gene expression profiles and signaling mechanisms in α2B-adrenoceptor-evoked proliferation of vascular smooth muscle cells
Source: BMC Syst Biol. 2017 Jun 28;11:65. doi: 10.1186/s12918-017-0439-8 (PMC5490158; doi:10.1186/s12918-017-0439-8)
Supplement: Supplementary file 6 — Top ten pathways indicated from kinase activity data significantly changed (−log(p) > 4) a t = 30 min (A) and at t = 24 h (B). (DOCX 195 kb) [file 12918_2017_439_MOESM6_ESM.docx]

**Additional file 6.**

Top ten pathways indicated from kinase activity data significantly changed (-log(p) > 4) a t = 30 min (A) and at t = 24 h (B). Pathways were identified for the following combined data sets: PTK_t=30min_ + STK_t=30min_ and PTK_t=24h_ + STK_t=24h_. Stars indicate pathways that are biased by overrepresentation on PamChips.

A. B.
