# Supplementary material for: Gene expression profiles and signaling mechanisms in α2B-adrenoceptor-evoked proliferation of vascular smooth muscle cells
Source: BMC Syst Biol. 2017 Jun 28;11:65. doi: 10.1186/s12918-017-0439-8 (PMC5490158; doi:10.1186/s12918-017-0439-8)

**Additional file 7.**

Putative PTK and STK upstream kinases at t = 30 min (A = PTK, B = STK) and t = 24 h (C = PTK, D = STK). Upstream kinases are ranked according to their determined specificity scores; red color indicates more specific kinases and less specific kinases are indicated with a color scale ranging from white to grey and black. The proportion of phosphorylated target peptides per total number of target peptides on the chip affects the specificity of the putative upstream kinases; the more of a kinase’s target peptides are phosphorylated in the array, the more specific the kinase is.

A.


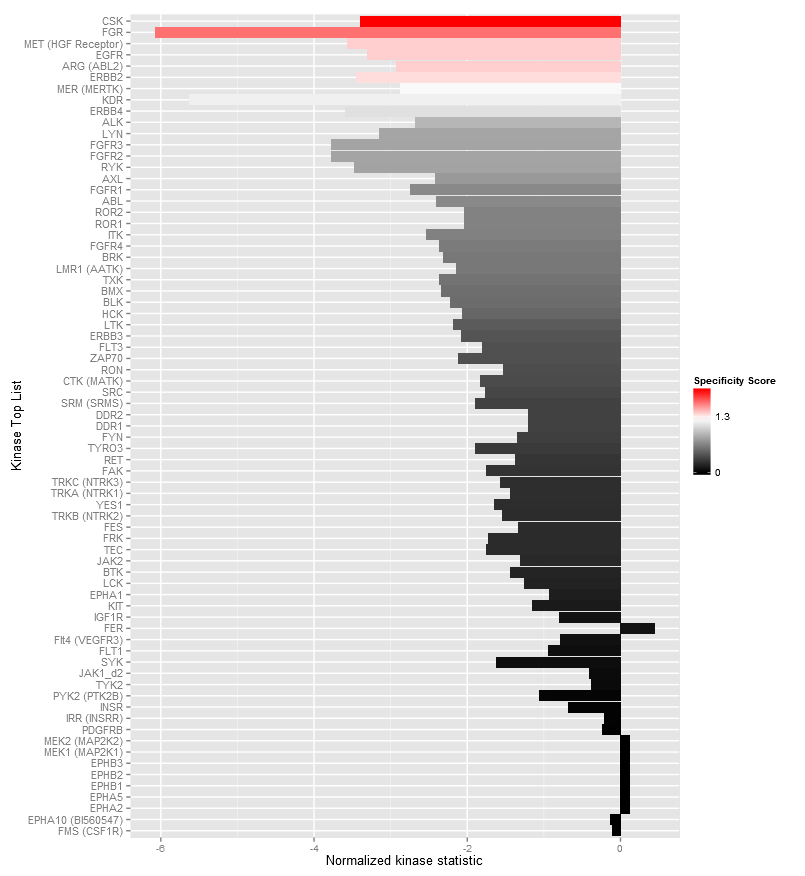


B.


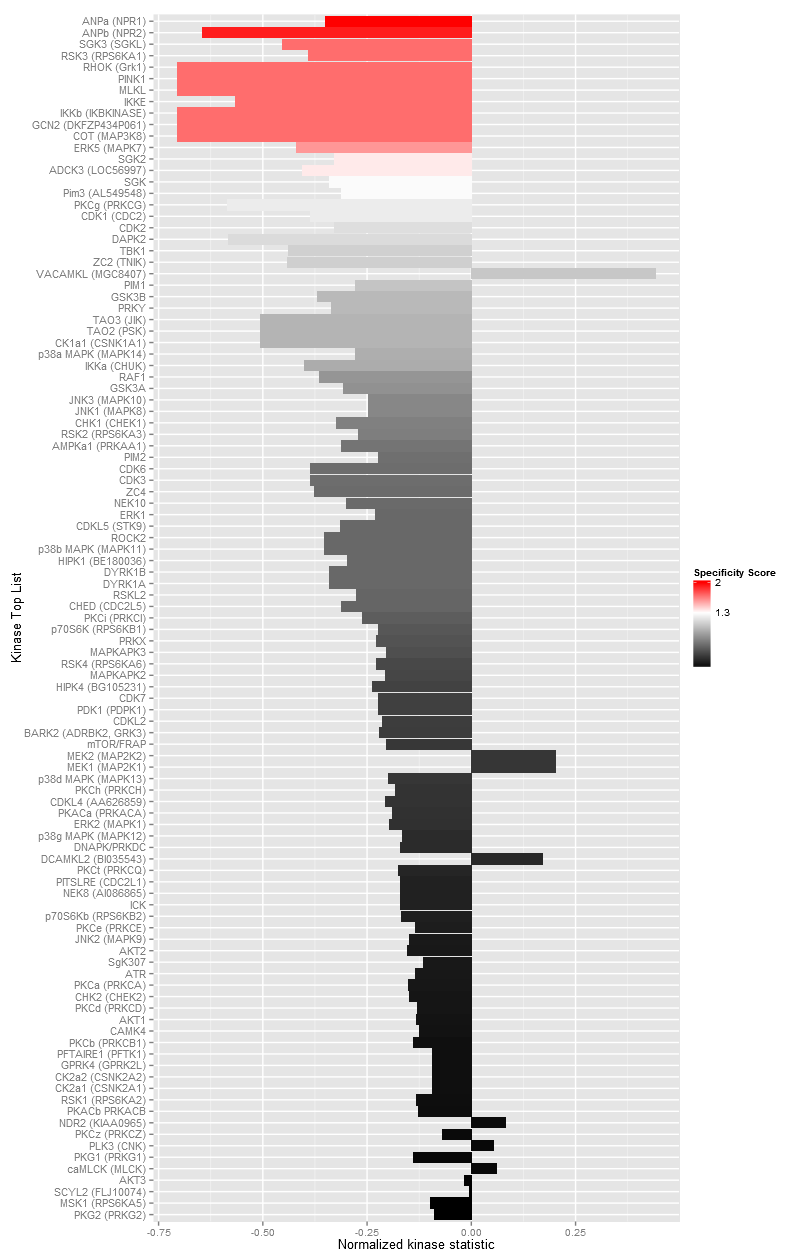


C.


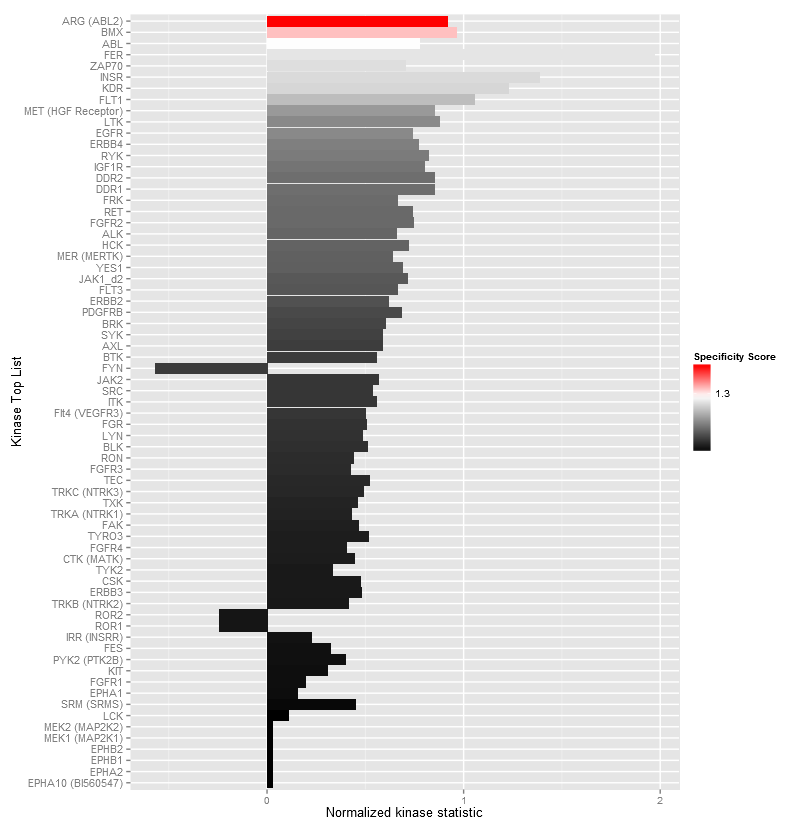


D.


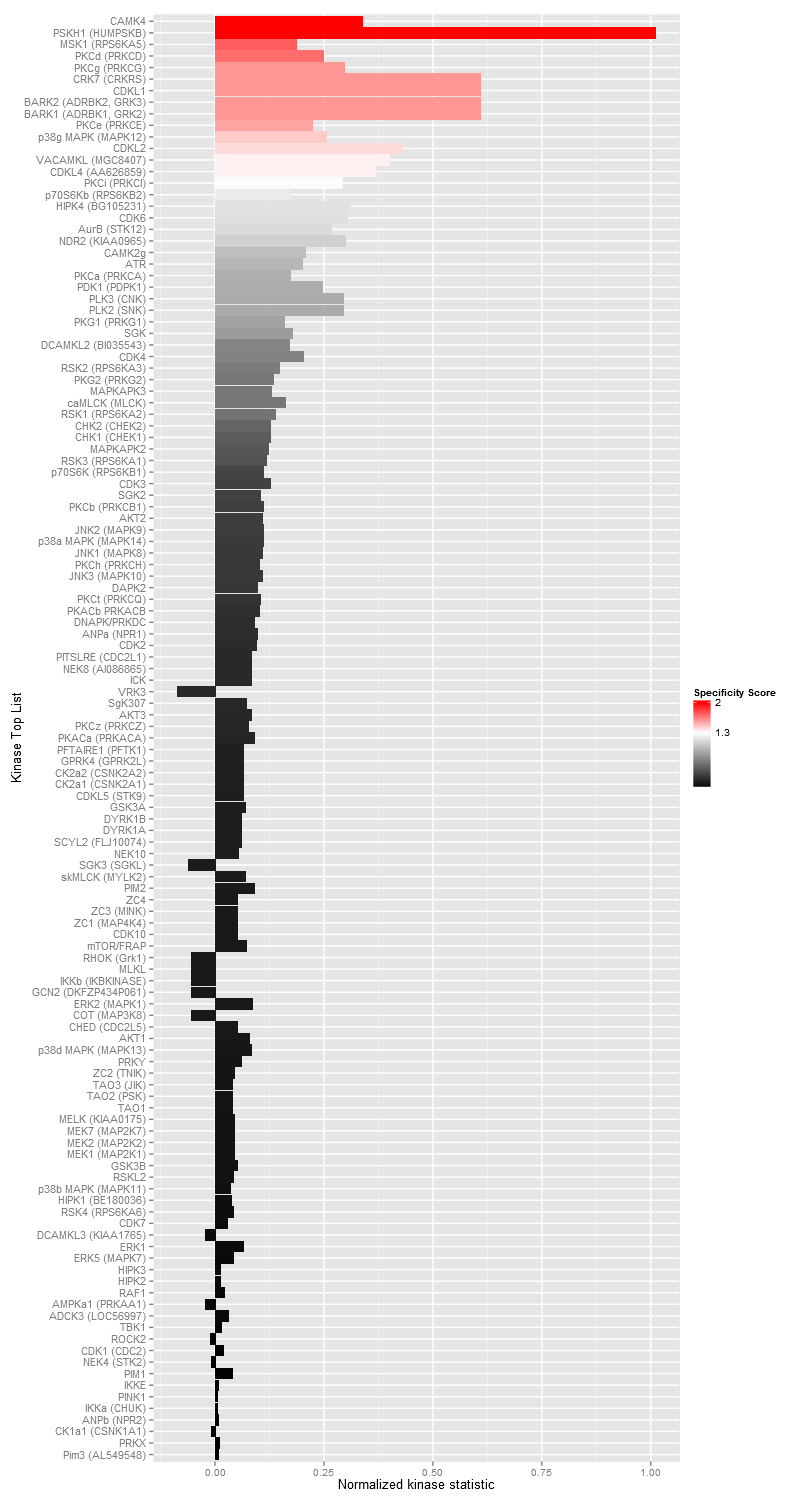

Supplement: Supplementary file 7 — Putative PTK and STK upstream kinases at t = 30 min (A = PTK, B = STK) and t = 24 h (C = PTK, D = STK). (DOCX 247 kb) [file 12918_2017_439_MOESM7_ESM.docx]
