# Supplementary material for: Gene expression profiles and signaling mechanisms in α2B-adrenoceptor-evoked proliferation of vascular smooth muscle cells
Source: BMC Syst Biol. 2017 Jun 28;11:65. doi: 10.1186/s12918-017-0439-8 (PMC5490158; doi:10.1186/s12918-017-0439-8)
Supplement: Supplementary file 9 — Time-lapse video of wildtype A7r5 vascular smooth muscle cell contraction induced with 100 nM vasopressin perfusion. (PPTX 5531 kb) [file 12918_2017_439_MOESM9_ESM.pptx]

## Slide 1
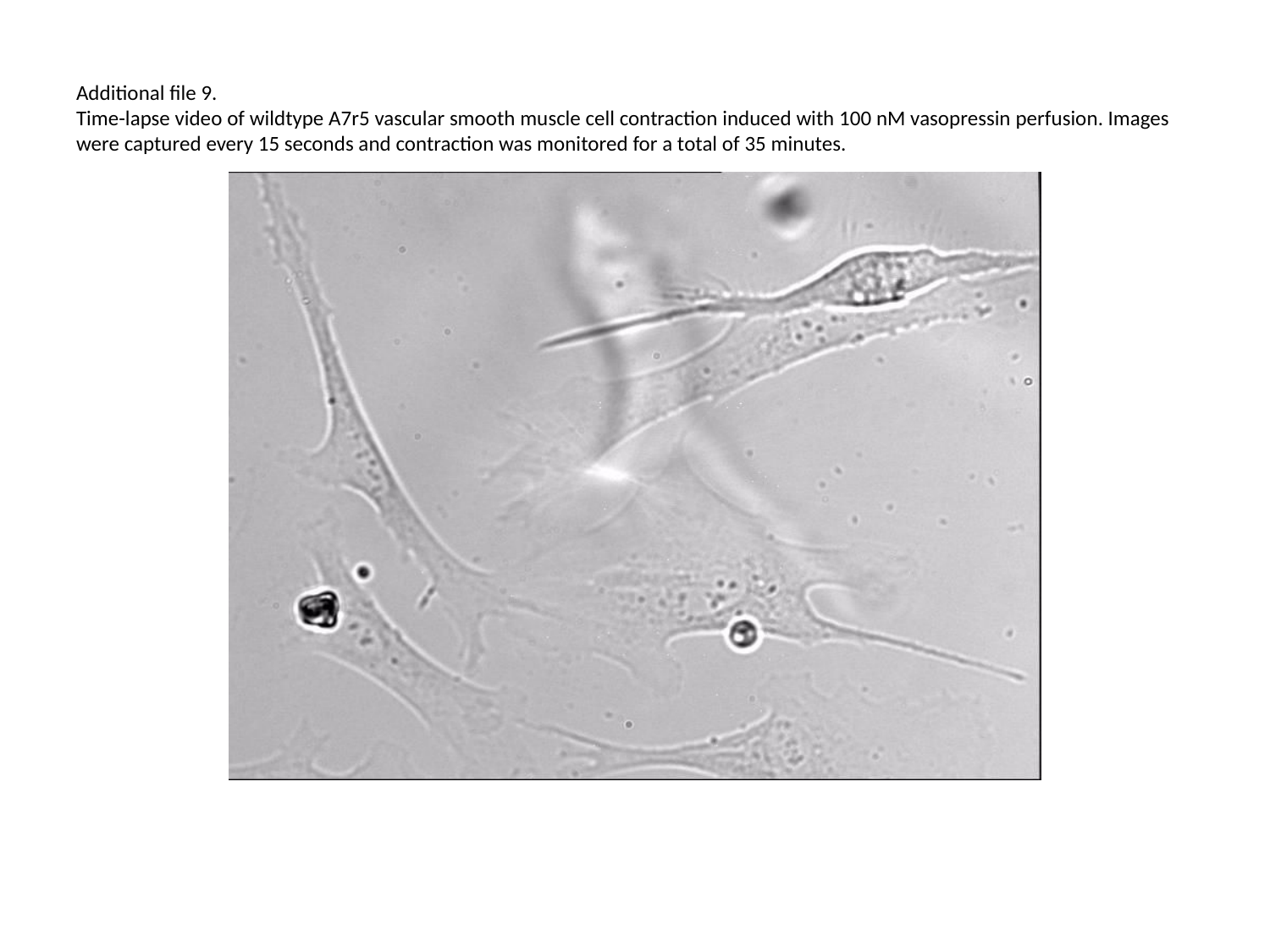

# Additional file 9. Time-lapse video of wildtype A7r5 vascular smooth muscle cell contraction induced with 100 nM vasopressin perfusion. Images were captured every 15 seconds and contraction was monitored for a total of 35 minutes.
