# Supplementary material for: Gene expression profiles and signaling mechanisms in α2B-adrenoceptor-evoked proliferation of vascular smooth muscle cells
Source: BMC Syst Biol. 2017 Jun 28;11:65. doi: 10.1186/s12918-017-0439-8 (PMC5490158; doi:10.1186/s12918-017-0439-8)

**Additional file 11.** Immune response_Fc epsilon RI pathway. Blue thermometers indicate inhibition of kinase activity.


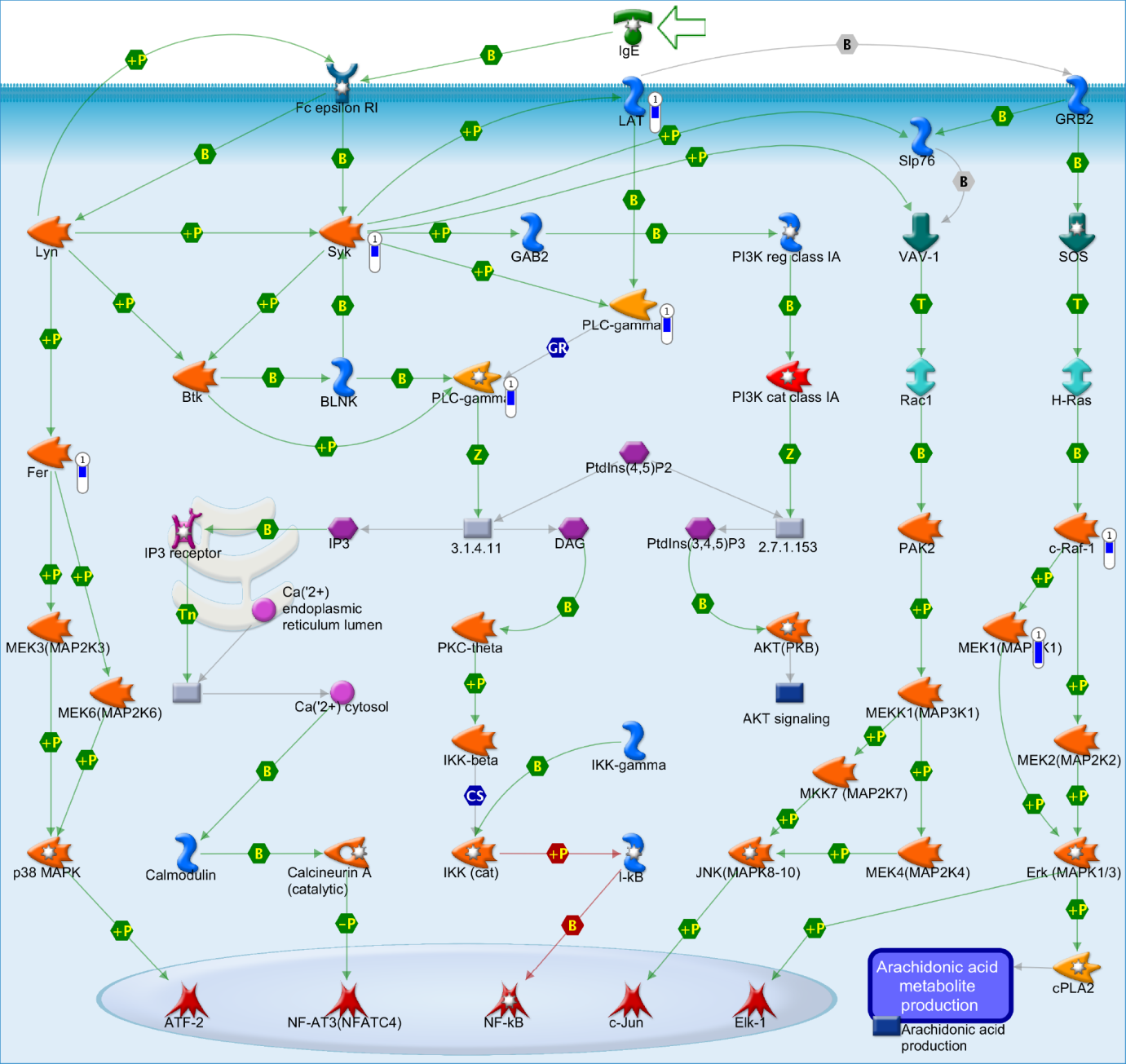

Supplement: Supplementary file 11 — Immune response_Fc epsilon RI pathway. Blue thermometers indicate inhibition of kinase activity. (DOCX 666 kb) [file 12918_2017_439_MOESM11_ESM.docx]
